# Protein/AS01_B_ vaccination elicits stronger, more Th2-skewed antigen-specific human T follicular helper cell responses than heterologous viral vectors

**DOI:** 10.1016/j.xcrm.2021.100207

**Published:** 2021-02-22

**Authors:** Carolyn M. Nielsen, Ane Ogbe, Isabela Pedroza-Pacheco, Susanne E. Doeleman, Yue Chen, Sarah E. Silk, Jordan R. Barrett, Sean C. Elias, Kazutoyo Miura, Ababacar Diouf, Martino Bardelli, Rebecca A. Dabbs, Lea Barfod, Carole A. Long, Barton F. Haynes, Ruth O. Payne, Angela M. Minassian, Todd Bradley, Simon J. Draper, Persephone Borrow

**Affiliations:** 1 Jenner Institute, University of Oxford, Oxford, UK; 2 Nuffield Department of Clinical Medicine, University of Oxford, Oxford, UK; 3 Human Vaccine Institute, Duke University School of Medicine, Duke University, Durham, NC, USA; 4 Laboratory of Malaria and Vector Research, NIAID/NIH, Rockville, MD, USA

**Keywords:** T follicular helper cells, Tfh cells, vaccines, malaria, Th1, Th2, clinical trials, heterologous viral vectors, AS01, antibody, adaptive immunity

## Abstract

Interactions between B cells and CD4^+^ T follicular helper (Tfh) cells are key determinants of humoral responses. Using samples from clinical trials performed with the malaria vaccine candidate antigen *Plasmodium falciparum* merozoite protein (PfRH5), we compare the frequency, phenotype, and gene expression profiles of PfRH5-specific circulating Tfh (cTfh) cells elicited by two leading human vaccine delivery platforms: heterologous viral vector prime boost and protein with AS01_B_ adjuvant. We demonstrate that the protein/AS01_B_ platform induces a higher-magnitude antigen-specific cTfh cell response and that this correlates with peak anti-PfRH5 IgG concentrations, frequency of PfRH5-specific memory B cells, and antibody functionality. Furthermore, our data indicate a greater Th2/Tfh2 skew within the polyfunctional response elicited following vaccination with protein/AS01_B_ as compared to a Th1/Tfh1 skew with viral vectors. These data highlight the impact of vaccine platform on the cTfh cell response driving humoral immunity, associating a high-magnitude, Th2-biased cTfh response with potent antibody production.

## Highlights

CD4 Tfh comparison in malaria vaccine trials using leading human vaccine platforms
Protein/AS01_B_ drives stronger antigen-specific Tfh responses than viral vectors
Greater T(f)h2 skewing of antigen-specific CD4 T cells in protein/AS01_B_ vaccinees
Antigen-specific CD4 T(fh) cell parameters correlate with functional antibody

## Graphical abstract

**Figure:**
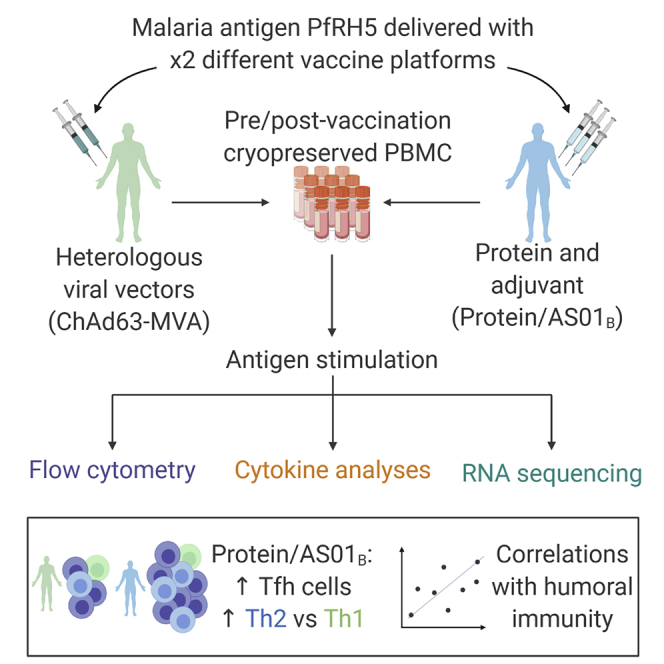

## Teaser

Nielsen et al. evaluate the induction of antigen-specific T follicular helper cell responses in human malaria vaccine trials using protein/adjuvant (AS01_B_) or heterologous viral vector platforms. They demonstrate that protein/AS01_B_ drives a higher-magnitude polyfunctional response with greater skewing toward a Th2 phenotype, associated with superior production of neutralizing antibodies.

## Introduction

For many pathogens, protective immunity is conferred by antibodies. A successful vaccine is therefore one that is capable of driving a B cell response that ultimately results in the production of sufficient circulating antibody to neutralize the pathogen and/or block disease development in future infection. Antibody concentration requirements for protection are dependent on pathogen-specific variables, and some targets, such as the blood-stage *Plasmodium falciparum* malaria parasites, have life cycle characteristics that necessitate a very robust antibody response. It is therefore of great interest to understand how vaccine antigens can be formulated and delivered to maximize humoral immunogenicity. Here, we focus on the impact of vaccine platform on the immune response elicited in humans by comparing 2 leading vaccine platforms that were used to deliver the same blood-stage *P*. *falciparum* malaria antigen RH5 (PfRH5) in Phase Ia trials: heterologous viral vectors (chimpanzee adenovirus serotype 63 prime, followed by modified vaccinia virus Ankara boost [henceforth referred to as ChAd63-MVA]^1^) and 3-dose full-length PfRH5 protein co-administered with the GlaxoSmithKline adjuvant AS01_B_ (protein/AS01_B_^2^). These 2 platforms have been used in vaccine development for a diverse range of diseases, including Ebola (heterologous viral vectors), pre-erythrocytic malaria (RTS,S/ Mosquirix), and shingles (Shingrix; both AS01; reviewed in O’Donnell and Marzi,^3^ Laurens,^4^ and Heinemann et al.^5^). Primary analysis of the responses elicited in each trial demonstrated that while the ChAd63-MVA heterologous viral vector platform elicited a PfRH5-specific T cell interferon-γ (IFN-γ) response >2-fold higher than that stimulated by the protein/AS01_B_ regimen (Mann-Whitney, p = 0.0019; Table 1), the anti-PfRH5 immunoglobulin G (IgG) concentration induced by the protein/AS01_B_ regimen was almost 10-fold higher than that elicited by the ChAd63-MVA heterologous viral vector platform (Mann-Whitney, p < 0.0001; Table 1; A.M.M. et al., data not shown).^1^ This highlighted a need for a more in-depth immunological comparison (particularly of differences in the CD4^+^ T follicular helper [Tfh] cell response) between these platforms in their entirety, as they would realistically be deployed, to elucidate their mechanism of action and guide platform selection for future pathogen-specific vaccines.

**Table 1:** ChAd63-MVA and protein/AS01_B_ clinical trials

Vaccine platform		Clinical trials						Subset of vaccinees used in analyses					
		n		Vaccination time points				n		Vaccinee demographics		ELISA and ELISpot responses	
				Day 0	Day 28	Day 56	Day 182			Median age, y (range)	Female, %	Mean day 84 anti-RH5 IgG (range) μg/mL	Mean day 140 IFN-γ ELISpot (range) SFU/million PBMCs
Heterologous viral vectors ChAd63 (prime) and MVA (boost) expressing PfRH5 (NCT02181088) ChAd63-MVA		4	24	5 × 10^9^ vp ChAd63-RH5	–	–	–	–					
		4		5 × 10^10^ vp ChAd63-RH5	–	–	–						
		8		5 × 10^10^ vp ChAd63-RH5	–	1 × 10^8^ PFU MVA-RH5	–	8	15^a^	25 (18–48)	47	7 (0.5–17.5)	681 (66.7–1,649.0)
		8		5 × 10^10^ vp ChAd63-RH5	–	2 × 10^8^ PFU MVA RH5	–	7					
Full-length PfRH5 protein (RH5.1) with AS01_B_ adjuvant (NCT02927145) protein/AS01_B_	Phase Ia	12	59	2 μg RH5 + AS01_B_	2 μg RH5 + AS01_B_	2 μg RH5 + AS01_B_	–	9	57^a^	28 (18–46)	67	67 (22.5–209.3)	318 (10.7–1,256.0)
		12		10 μg RH5 + AS01_B_	10 μg RH5 + AS01_B_	10 μg RH5 + AS01_B_	–	11					
		14		50 μg RH5 + AS01_B_	50 μg RH5 + AS01_B_	50 μg RH5 + AS01_B_	–	10					
		12		50 μg RH5 + AS01_B_	50 μg RH5 + AS01_B_	–	10 μg RH5 + AS01_B_	10^b^					
	Phase IIa	17		10 μg RH5 + AS01_B_	10 μg RH5 + AS01_B_	10 μg RH5 + AS01_B_	–	17					

ELISA and ELISpot data refer to standard regimen only. PFU, plaque-forming unit; SFU, spot-forming unit; vp, viral particle.

a Dose groups are pooled for all assays and analyses (see STAR methods).

b Samples after the delayed fractional dose at day 182 are not included in the present study, with the exception of 1 sample for RNA-seq. ELISA and ELISpot data refer to standard regimen only.

Through the provision of help to B cells in germinal centers (GCs), Tfh cells act as critical orchestrators of humoral immunity and are key determinants of both B cell and antibody immunokinetics, controlling antibody affinity maturation in GCs and influencing plasma cell versus memory B cell (mBC) fates (reviewed in Inoue et al.^6^ and Vinuesa et al.^7^) While the origin of peripheral Tfh cells remains unclear, multiple studies suggest that circulating (c)Tfh cells exhibit phenotypic, functional, gene expression, and T cell receptor repertoire profiles similar to those of lymphoid tissue Tfh populations,^8^, ^9^, ^10^ and constitute valid proxies for providing insight into GC responses.^11^, ^12^, ^13^ For example, cTfh cells can provide help to B cells in *in vitro* co-culture systems,^8^^,^^14^ and within both peripheral blood and GC populations, the Tfh2 and Tfh17 cell subsets are more capable of driving B cell differentiation and antibody secretion than Tfh1 cells (reviewed in Koutsakos et al.^15^).

Given the constraints on studying GC responses imposed by the limited availability of lymphoid tissue samples from clinical trials, there has been substantial interest in the last decade in determining the relationship between cTfh cell responses and humoral immunity, with the longer-term goal of characterizing the attributes of the Tfh cell response that can drive a desired type of antibody response. Multiple studies have observed increases in activated cTfh frequencies following vaccination.^9^^,^^12^^,^^16^, ^17^, ^18^, ^19^, ^20^, ^21^, ^22^, ^23^, ^24^, ^25^, ^26^, ^27^, ^28^ However, to date there have not been any in-depth analyses of the impact of the vaccine platform on the magnitude, kinetics, and quality of the antigen-specific cTfh cell response and its relationship to the humoral response.

Here, alongside extensive *ex vivo* analyses of changes in global cTfh populations, we used an adapted activation-induced marker (AIM) assay (A.O., data not shown)^21^^,^^29^ to compare the frequencies of PfRH5-specific cTfh cells elicited following delivery of the PfRH5 antigen using ChAd63-MVA or protein/AS01_B_ vaccine platforms. Significant increases in circulating PfRH5-specific cTfh cells were induced following vaccination in each trial, but both the total memory CD4^+^ T cell response and total memory cTfh cell response elicited by the protein/AS01_B_ platform were of higher magnitude than those elicited by the heterologous viral vectors. Notably, PfRH5-specific cTfh frequencies correlated with readouts of humoral immunogenicity, including *in vitro* anti-malarial growth inhibition activity (GIA) by polyclonal post-vaccination anti-PfRH5 IgG, a defined mechanistic correlate for vaccine-induced protection against blood-stage malaria.^30^^,^^31^ We furthermore provide phenotypic, cytokine, and transcriptomic data to suggest that the protein/AS01_B_ platform also drove a response skewed more toward Th2/Tfh2 than that seen with the ChAd63-MVA platform, which is consistent with a role for Tfh2 cells in providing greater B cell help (reviewed in Koutsakos et al.^15^). Our analysis focuses on comparison of the responses elicited by the 2 vaccine platforms after completion of the full 2-dose (viral vectors) or 3-dose (protein/AS01_B_) immunization regimen, but significant quantitative and qualitative differences were also observed after the first dose of each vaccine had been administered. Insight into how vaccination strategies can be modulated to optimize B cell responses is of great interest for the blood-stage malaria vaccine development field, as well as for other prophylactic vaccine development programs in which humoral immunity is critical, including for viruses such as human immunodeficiency virus type 1 (HIV-1). At present, there are no validated approaches for increasing the production or function of Tfh cells in humans. Our demonstration of the influence of the vaccine platform on both the magnitude and quality of the antigen-specific cTfh response elicited in human vaccine recipients therefore constitutes an important step forward in informing the design of vaccination strategies that will elicit robust Tfh support for the generation of protective pathogen-specific humoral responses.

## Results

### Differences in the total post-vaccination T cell responses activated by ChAd63-MVA and protein/AS01_B_ vaccine platforms

**Figure 1:**
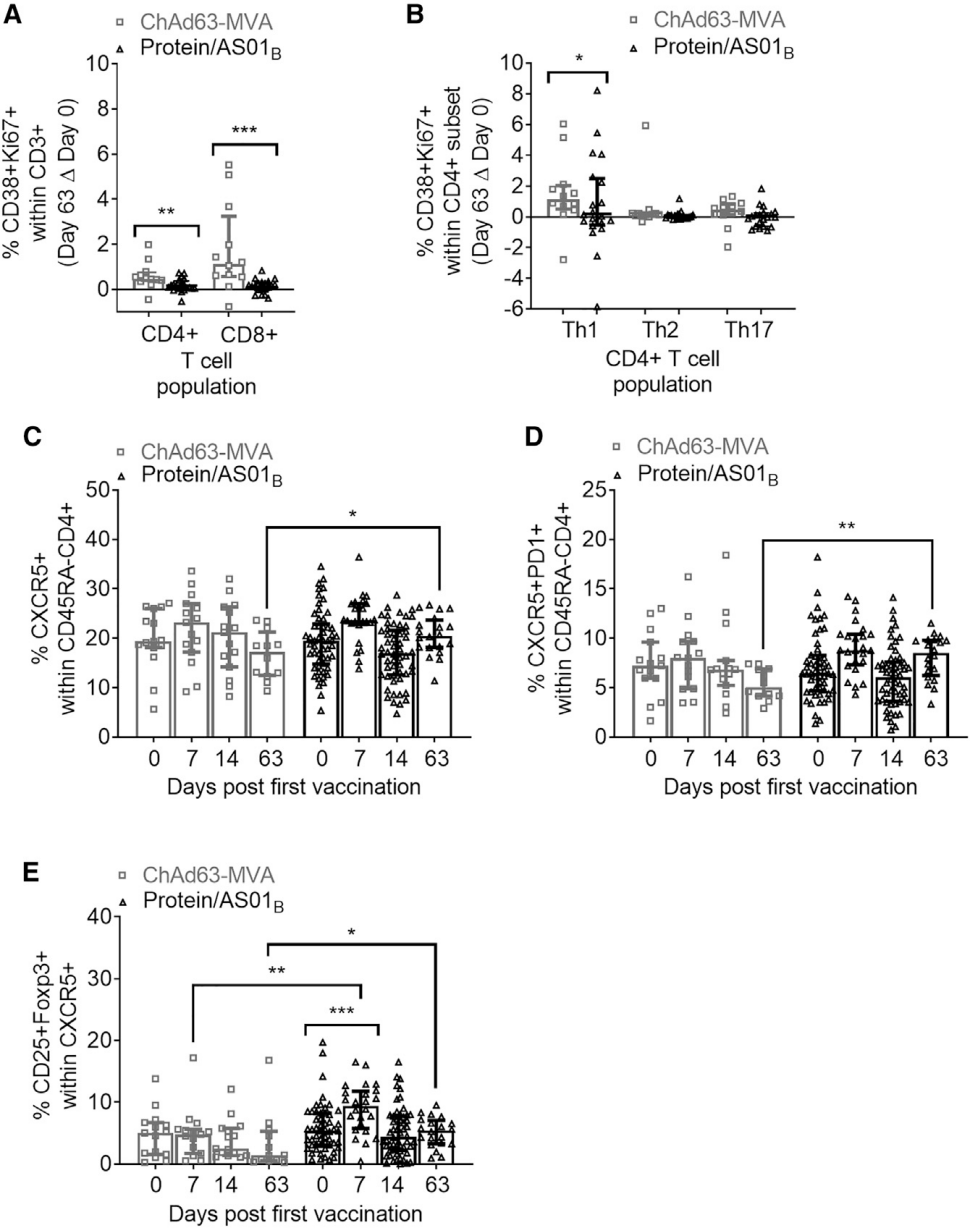
PfRH5 vaccination with the ChAd63-MVA platform induces greater activation of total circulating CD8^+^ and CD4^+^ T cell populations, while the protein/AS01_B_ platform elicits a more sustained increase in cTfh-phenotype cells PBMCs from days 0, 7, 14, and 63 were stained *ex vivo* and analyzed using the gating strategies shown in Figures S1–S3. (A and B) Increases at day 63 in activated CD38^+^Ki67^+^ cells within total CD8^+^ and CD4^+^ T cells (A) or Th1 (CXCR3^+^CCR6^−^), Th2 (CXCR3^−^CCR6^−^), and Th17 (CXCR3^−^CCR6^+^) CD4^+^ T cell subsets (B) were compared between platforms following subtraction of day 0 CD38^+^Ki67^+^ frequencies in paired samples. (C–E) Frequencies of total cTfh cells defined as CXCR5^+^ (C) or CXCR5^+^PD1^+^ (D) cells within the CD45RA^−^CD4^+^ T cell population were compared between platforms and the frequency of cTfr cells within total cTfh cells (defined as the CD25^+^Foxp3^+^ subset) (E). All of the available samples are plotted (ChAd63-MVA/protein/AS01_B_): day 0, n = 15/54; day 7, n = 15/24; day 14, n = 15/54; day 63, n = 12/20. For intra-trial comparisons (E), only vaccinees with all 4 time points were analyzed: ChAd63-MVA, n = 12, protein/AS01_B_, n = 17. Comparisons were performed with Mann-Whitney tests (between trials) or Friedman tests with Dunn’s correction for multiple comparisons (within trials comparing day 0 to post-vaccination time points). ∗p < 0.5, ∗∗p < 0.01, and ∗∗∗p < 0.001. In all of the panels, each point represents a vaccinee. Bars and lines denote medians and interquartile ranges, respectively.

We first used multiparameter flow cytometry to assess activation—as measured by CD38/Ki67 co-expression—of CD4^+^ and CD8^+^ T cells within the CD3^+^ lymphocyte population, as well as of Th1/Th2/Th17 subsets within CD4^+^ T cells, using the gating strategy shown in Figure S1. Consistent with prior observations that viral vector platforms are effective drivers of T cell responses (Table 1; e.g., as reviewed for malaria vaccines in Ewer et al.^32^ and Sebastian and Gilbert^33^), after subtracting baseline *ex vivo* CD38^+^Ki67^+^ expression (day 0) from values at day 63 (1 week post-final vaccination), we observed a greater increase in the frequency of CD38^+^Ki67^+^ CD4^+^ and CD8^+^ T cells in the ChAd63-MVA vaccinees as compared to the protein/AS01_B_ vaccinees (Figure 1A, p = 0.0074 and p = 0.0005, respectively). Within the CD4^+^ T cell population, we used the same approach to further assess the activation of Th1 (CXCR3^+^CCR6^−^), Th2 (CXCR3^−^CCR6^−^), and Th17 (CXCR3^−^CCR6^+^) subsets. The activation of Th1-phenotype CD4^+^ T cells was significantly greater in the ChAd63-MVA vaccinees (Figure 1B, p = 0.0386), indicating that this platform elicited a more robust level of total (i.e., not necessarily PfRH5-specific) T cell activation.

Next, the frequency of cTfh cells within the memory (CD45RA^−^) CD4^+^ T cell population was compared at baseline, 1 and 2 weeks (days 7 and 14) after the first vaccination, and at day 63. cTfh cells were defined as either CXCR5^+^ or CXCR5^+^PD1^+^ cells within CD45RA^−^CD4^+^ T cells (Figure S2), consistent with 2 commonly used approaches.^8^^,^^10^, ^11^, ^12^, ^13^, ^14^^,^^18^^,^^20^^,^^23^^,^^27^ Within both trials there was a non-significant trend toward an increase in cTfh cells at day 7 (Figures 1C and 1D). A similar trend was also observed at day 63 in the protein/AS01_B_ but not ChAd63-MVA vaccinees; hence, day 63 cTfh frequencies in protein/AS01_B_ vaccinees were significantly higher than those in ChAd63-MVA vaccinees (CXCR5^+^, p = 0.0428; CXCR5^+^PD1^+^, p = 0.0015).

Using the CXCR5^+^ definition of cTfh cells, we then assessed the activation of cTfh cells post-vaccination through the measurement of the co-expression of CD38/Ki67, ICOS/Ki67 or ICOS/PD1, or ICOS alone (Figure S2; ICOS/Ki67 not shown, as no changes were detected). Limited indications of activation were detectable in both trials at day 7 through an increase in the frequency of CD38^+^Ki67^+^ (ChAd63-MVA vaccinees; p = 0.0049) or ICOS^+^PD1^+^ and ICOS^+^ (protein/AS01_B_ vaccinees; ICOS^+^PD1^+^, p = 0.0196; ICOS^+^, p = 0.0123) Tfh cells.

We also assessed post-vaccination changes in the frequency of circulating subsets of regulatory CD4^+^ T cells (Tregs: the CD25^+^Foxp3^+^ subset of CD4^+^ T cells; T follicular regulatory [cTfr]-phenotype cells: CD25^+^Foxp3^+^ subset of CD4^+^CXCR5^+^ T cells). While no significant differences in the frequency of Tregs within the total CD4^+^ T cell population were observed within or between trials at any time point (Figure S3), there was a highly significant increase in cTfr-phenotype cells between days 0 and 7 within the protein/AS01_B_ vaccinees (p = 0.0001), and the frequency at day 7 was significantly greater than that of ChAd63-MVA vaccinees (p = 0.0017; Figure 1E). The trends observed in Figures 1C and 1D persist even when Treg cells are excluded (data not shown).

Given the importance of ICOS in CD4^+^ Tfh cell differentiation^34^ and recent reports suggesting that cTfr-phenotype cells may contribute to the provision of GC help for B cells (reviewed in Xie and Dent^35^), these observations may be indicative of the induction of more robust T cell-mediated help for B cells by the protein/AS01_B_ platform.

### The protein/AS01_B_ platform elicits a higher-frequency antigen-specific CD4^+^ T cell response and induces a greater Th2 bias in the responding cells

**Figure 2:**
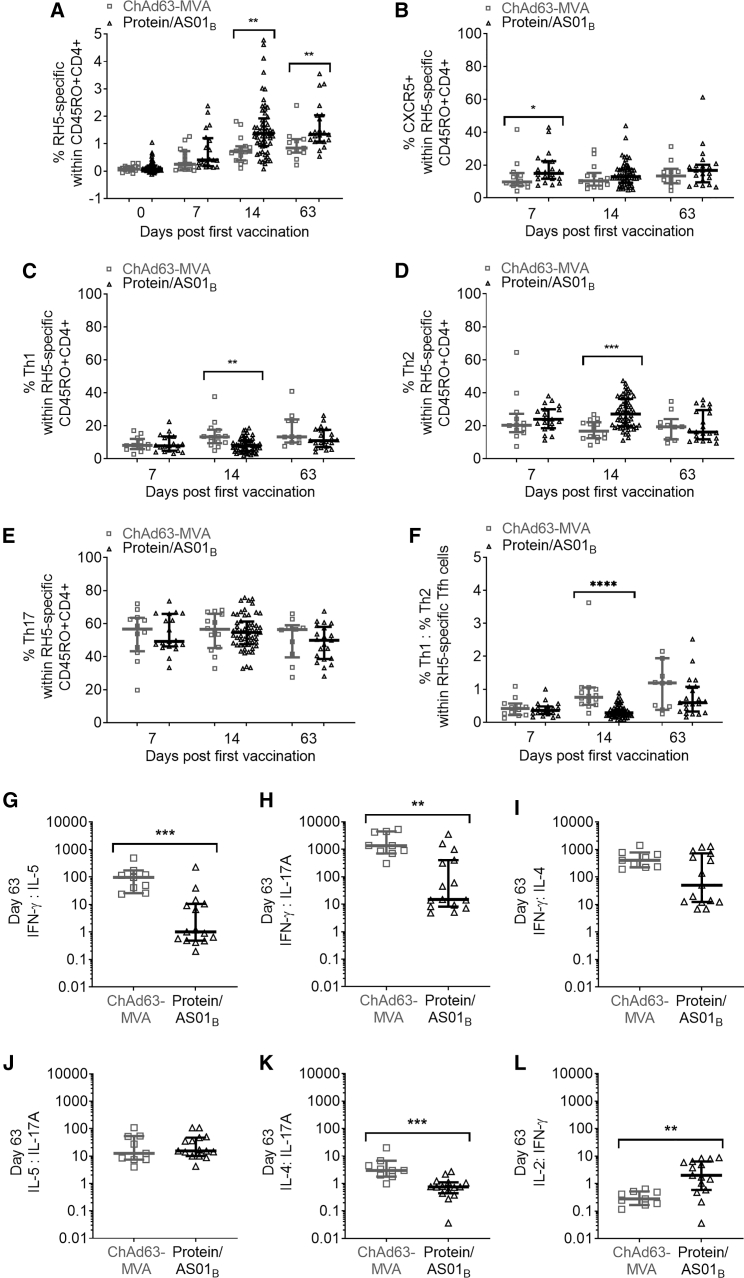
Protein/AS01_B_ platform induces a more robust and Th2-skewed PfRH5-specific CD4^+^ response than ChAd63-MVA vaccination PBMCs from days 0, 7, 14, and 63 were stimulated with medium alone or a PfRH5 peptide pool for 24 h, then stained and analyzed, identifying PfRH5-specific cells as those co-expressing CD25 with OX40, CD137, or CD69 following stimulation. Frequencies of CXCR5^+^ (cTfh cells), Th1 (CXCR3^+^CCR6^−^), Th2 (CXCR3^−^CCR6^−^), and Th17 (CXCR3^−^CCR6^+^) were also quantified within RH5-specific CD4^+^CD45RO^+^ cells (all gating as in Figure S4). (A) Frequencies of PfRH5-specific cells within CD45RO^+^CD4^+^ T cells were compared between each time point between trials. (B–F) Within the PfRH5-specific CD45RO^+^CD4^+^ T cell population, the proportion of cells that were CXCR5^+^ cTfh (B), CXCR3^+^CCR6^−^ (Th1) (C), CXCR3^−^CCR6^−^ (Th2) (D), CXCR3^−^CCR6^+^ (Th17) (E), or the ratio of Th1:Th2 cells (F) was also compared between platforms at each time point. (G–L) A multiplex bead-based assay was then used to measure the supernatant concentrations of cytokines, and the Th1/Th2/Th17 skew of the cytokine response was determined by calculating ratios of IFN-γ:IL-5 (G), IFN-γ:IL-17A (H), IFN-γ:IL-4 (I), IL-5-IL-17A (J), IL-4:IL-17A (K), and IL-2:IFN-γ (L) in supernatants from day 63 PBMCs. In (A), all of the available samples were analyzed (ChAd63-MVA/protein/AS01_B_): day 0, n = 15/57; day 7, n = 15/20; day 14, n = 15/57; and day 63, n = 11/22. In (B–F), samples were excluded if the total number of PfRH5-specific CD45RO^+^CD4^+^ T cells was <50 (ChAd63-MVA/protein/AS01_B_): day 7, n = 12/20; day 14, n = 14/56; and day 63, n = 10/21. A multiplex assay (G–L) was run on a subset of samples (ChAd63-MVA/protein/AS01_B_): day 63, n = 9/15. Comparisons between trials were performed using Mann-Whitney tests. ∗p < 0.05, ∗∗p < 0.01, ∗∗∗p < 0.001, and ∗∗∗∗p < 0.0001. In all of the panels, each point represents a vaccinee. Lines denote medians and interquartile ranges.

To extend the *ex vivo* analyses of global alterations in circulating T cell subsets, we used an *in vitro* AIM assay to identify PfRH5-specific T cells within the memory-phenotype CD4^+^ T cell population on the basis of co-expression of CD25 with OX40 and/or CD137 and/or CD69 following PfRH5 peptide pool stimulation (gating strategy shown in Figure S4; data not shown). In contrast to the observation of higher-magnitude increases in the overall levels of *ex vivo* CD4^+^ T cell activation in ChAd63-MVA vaccinees (Figure 1A), the AIM assay revealed that the PfRH5-specific CD4^+^ T cell response was in fact more robust in the protein/AS01_B_ vaccinees at both day 14 (p = 0.0021) and day 63 (p = 0.0057; Figure 2A). We also looked at the antigen-specific CD8^+^ T cell response, assessed based on the co-expression of CD25 with CD137 and/or CD69.^36^^,^^37^ Using this readout, both vaccine platforms induced a significant PfRH5-specific CD8^+^ T cell response at day 63 (Figure S4; ChAd63-MVA, p = 0.0001; protein/AS01_B_, p = 0.0004), and there were no significant differences between trials (Figure S4).

Within the PfRH5-specific CD4^+^ T cell population, we observed a trend toward a higher proportion of CXCR5^+^ (cTfh) cells in protein/AS01_B_ as compared to ChAd63-MVA vaccinees at all post-vaccination time points (Figure 2B), which was weakly significant at day 7 (p = 0.0471). Further phenotypic analysis of the PfRH5-specific CD4^+^ T cell population revealed a predominance of Th17 cells (CXCR3^−^CCR6^+^) in all vaccinees, but it identified a trend toward a higher proportion of Th1 cells (CXCR3^+^CCR6^−^) in the ChAd63-MVA platform vaccinees (Figure 2C) and a higher proportion of Th2 cells (CXCR3^−^CCR6^−^) in the protein/AS01_B_ vaccinees (Figure 2D), both of which reached statistical significance at day 14 post-vaccination (p = 0.0015 and p = 0.0002, respectively). There were no significant differences at any post-vaccination time point in the proportion of Th17 cells (CXCR3^−^CCR6^+^) within the PfRH5-specific memory CD4^+^ T cell population (Figure 2E). In light of this apparent divergence in the contribution of Th1 and Th2 cells between platforms, we also compared the ratio of Th1:Th2 cells within the vaccine-induced RH5-specfic CD45RO^+^CD4^+^ T cell population and detected a higher ratio in the ChAd63-MVA vaccinees (Figure 2F; day 14, p < 0.0001).

To complement the phenotypic analysis of the Th1/Th2/Th17 bias of PfRH5-specfic CD4^+^ T cells, we next measured the concentration of 13 different cytokines in supernatants of PfRH5 peptide pool-stimulated peripheral blood mononuclear cells (PBMCs) from the AIM assay. Ten of the 13 cytokines quantified were detected at significantly higher levels in the supernatants from PfRH5-stimulated cells at post-vaccination as compared to day 0 time points in one or both of the trials (Figure S5). Of these, interleukin-5 (IL-5), IL-9, IL-17A, and IL-22 concentrations were significantly higher in supernatants from protein/AS01_B_ vaccinees. Only IFN-γ and tumor necrosis factor-α (TNF-α) were higher in ChAd63-MVA vaccinees, although differences in the concentrations of these cytokines between trials did not reach statistical significance (Figure S5). To assess the skewing of the Th cytokines produced, we calculated ratios between supernatant concentrations of prototypical Th1, Th2, and Th17 cytokines. In line with the Th1 versus Th2 bias observed in the phenotype of the PfRH5-specific CD4^+^ T cells elicited in ChAd63 vaccinees, antigen-stimulated day 63 cell supernatants from these subjects had significantly higher Th1:Th2 (IFN-γ:IL-5; Figure 2G, p = 0.0002) and higher Th1:Th17 (IFN-γ:IL-17A; Figure 2H, p = 0.0021) cytokine ratios than those from protein/AS01_B_ vaccinees. Evaluation of the Th1:Th2 bias based on the IFN-γ:IL-4 ratio revealed a similar trend (ChAd63-MVA median ratio = 409.2; protein/AS01_B_ median ratio = 50.8), although the difference did not reach statistical significance (Figure 2I). There was no (IL-5:IL-17A) or minimal (IL-4:IL-17A, ChAd63-MVA median ratio = 3.0; protein/AS01_B_ median ratio = 0.7; p = 0.0001) difference between trials in the Th2:Th17 cytokine ratio (Figures 2J and 2K). Interestingly, the IL-2:IFN-γ ratio was higher in protein/AS01_B_ than ChAd63-MVA vaccinee supernatants (Figure 2L, p = 0.0027), potentially also indicating a greater Th1 bias in the latter group. All of the ratios calculated from day 14 supernatant cytokines were consistent with these trends (data not shown).

### The protein/AS01_B_ platform elicits a higher-magnitude, more Tfh2-biased antigen-specific cTfh cell response than ChAd63-MVA

**Figure 3:**
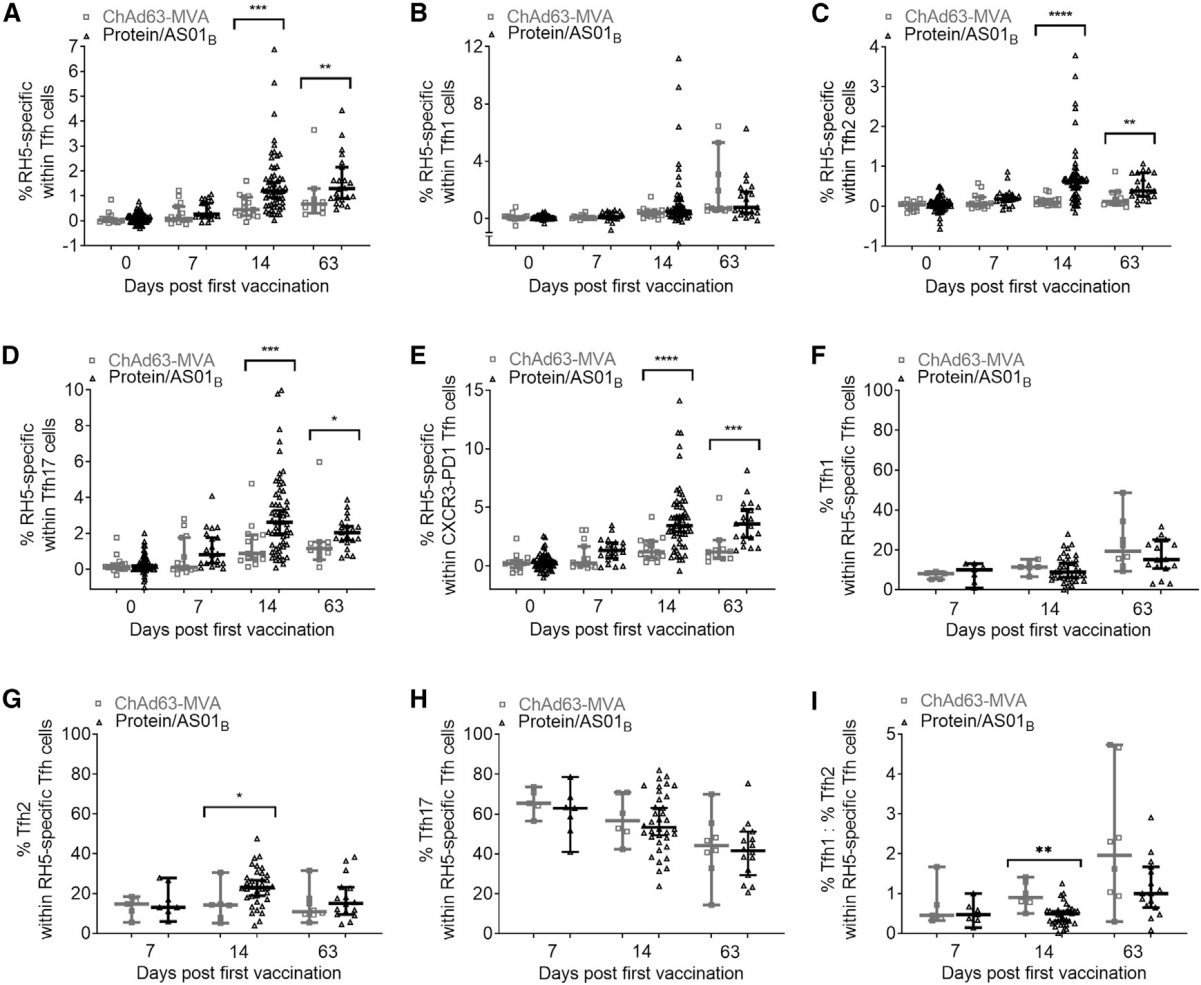
Protein/AS01_B_ platform induces a robust PfRH5-specific response within the cTfh cell population, with a qualitative skew toward Tfh2 PBMCs were stimulated as in Figure 2 with PfRH5-specific and Tfh1/2/17 gating as per Figures S4 and S6, respectively. cTfh cells were defined as CXCR5^+^ cells within CD45RO^+^CD4^+^ T cells and delineating the CXCR3-PD1^+^ subset of CXCR5^+^CD45RO^+^CD4^+^ cTfh. (A–E) Frequencies of PfRH5-specific cells were compared at each time point within cTfh cells (A), Tfh1 cells (B), Tfh2 cells (C), Tfh17 cells (D), and CXCR3^−^PD1^+^ cells (E). (F–I) Within the PfRH5-specific cTfh cell population, the proportion of cells at each time point that was Tfh1 cells (F), Tfh2 cells (G), Tfh17 cells (H), or the ratio of %Tfh1:Tfh2 (I) was also compared between platforms. All of the available samples were analyzed (ChAd63-MVA/protein/AS01_B_): day 0, n = 15/57; day 7, n = 15/20; day 14, n = 15/57; and day 63, n = 11/22. In (B–E), samples were excluded if the total number of Tfh1/Th2/Th17 or CXCR3^−^PD1^+^ cells was <50 in either the medium alone or PfRH5 peptide pool sample (ChAd63-MVA/protein/AS01_B_): day 0, n = 15/55–57; day 7, n = 15/19–20; day 14, n = 15/ 55–57; and day 63, n = 11/21. In (F–I), data points were excluded if the total number of responding cTfh cells was <50 (ChAd63-MVA/protein/AS01_B_): day 7, n = 5/7; day 14, n = 6/36; and day 63, n = 8/16. Comparisons between trials were performed using Mann-Whitney tests. ∗p < 0.05, ∗∗p < 0.01, ∗∗∗p < 0.001, and ∗∗∗∗p < 0.0001. In all of the panels, each point represents a vaccinee. Lines denote medians and interquartile ranges.

Following this finding that the protein/AS01_B_ platform elicited a higher-magnitude, more Th2-biased antigen-specific CD4^+^ T cell response than the ChAd63-MVA platform, we proceeded to use the AIM assay data to address the magnitude/nature of the PfRH5-specific cTfh cell responses elicited in each trial (defining cTfh cells as CXCR5^+^CD45RO^+^CD4^+^ cells and distinguishing subsets thereof within PfRH5-specific cells [identified as in Figure S4], as shown in Figure S6). Subpopulations of cTfh cells analyzed included the Tfh1-phenotype (CXCR3^+^CCR6^−^), Tfh2-phenotype (CXCR3^−^CCR6^−^), and Tfh17-phenotype (CXCR3^−^CCR6^+^) subsets, and also the CXCR3^−^PD1^+^ subpopulation of cTfh in which resting memory Tfh cells are most highly enriched.^9^ While both platforms induced detectable PfRH5-specific cTfh cell responses following vaccination (Figure S6), the protein/AS01_B_ platform elicited a substantially higher-magnitude PfRH5-specific cTfh response than ChAd63-MVA (Figure 3A), with statistically significant differences in antigen-specific frequencies within the cTfh cell subset at both day 14 (p = 0.0003) and day 63 (p = 0.0057). Differences at these time points remained significant when the magnitude of the antigen-specific cTfh cell response was instead compared in terms of the frequencies of PfRH5-specific cTfh cells within total CD45RO^+^CD4^+^ T cells (Figure S6). Intriguingly, PfRH5-specific responses varied between cTfh cell subpopulations; within the Tfh1-phenotype population, there was no significant difference between platforms in the frequency of PfRH5-specific cells (Figure 3B), but higher frequencies of PfRH5-specific cells were observed within the Tfh2-phenotype (Figure 3C) subset at day 14 (p < 0.0001) and day 63 (p = 0.0054) and the Tfh17-phenotype (Figure 3D) subset at day 14 (p = 0.0007) and day 63 (p = 0.0108) in protein/AS01_B_ versus ChAd63-MVA vaccinees. The former group also had a significantly higher frequency of PfRH5-specific cells within the CXCR3-PD1^+^ subset of cTfh cells (Figure 3E) at day 14 (p < 0.0001) and day 63 (p = 0.0002).

Within the total PfRH5-specific circulating cTfh population, there was a trend toward a higher proportion of Tfh1-phenotype cells in the ChAd63-MVA vaccinees (Figure 3F), and we also observed a higher proportion of Tfh2-phenotype cells in the protein/AS01_B_ vaccinees at day 14 (p = 0.0483; Figure 3G). The proportions of Tfh17-phenotype cells within the PfRH5-specific cTfh population (the majority of PfRH5-specific cells) were comparable between the 2 platforms at all time points (Figure 3H). These observations on the skew of the PfRH5-specific response parallel those made for the total memory CD4^+^ T cell population (Figure 2), and the ratio of Tfh1:Tfh2 cells within the RH5-specific Tfh cell population was higher in the ChAd63-MVA vaccinees (Figure 3I; day 14, p = 0.002). Notably, while we observed similar post-vaccination changes and differences between platforms in the parent CD4^+^ T cell population (Figure 2) and the cTfh subpopulation (Figure 3), the trends we reported for the former are not dependent on the inclusion of cTfh cells, as comparable differences were observed when CXCR5^+^ cells were excluded from these analyses (data not shown).

### Transcriptional profiling of PfRH5-specific CXCR5^+^ CD4 T cells from ChAd63-MVA and protein/AS01_B_ vaccinees

**Figure 4:**
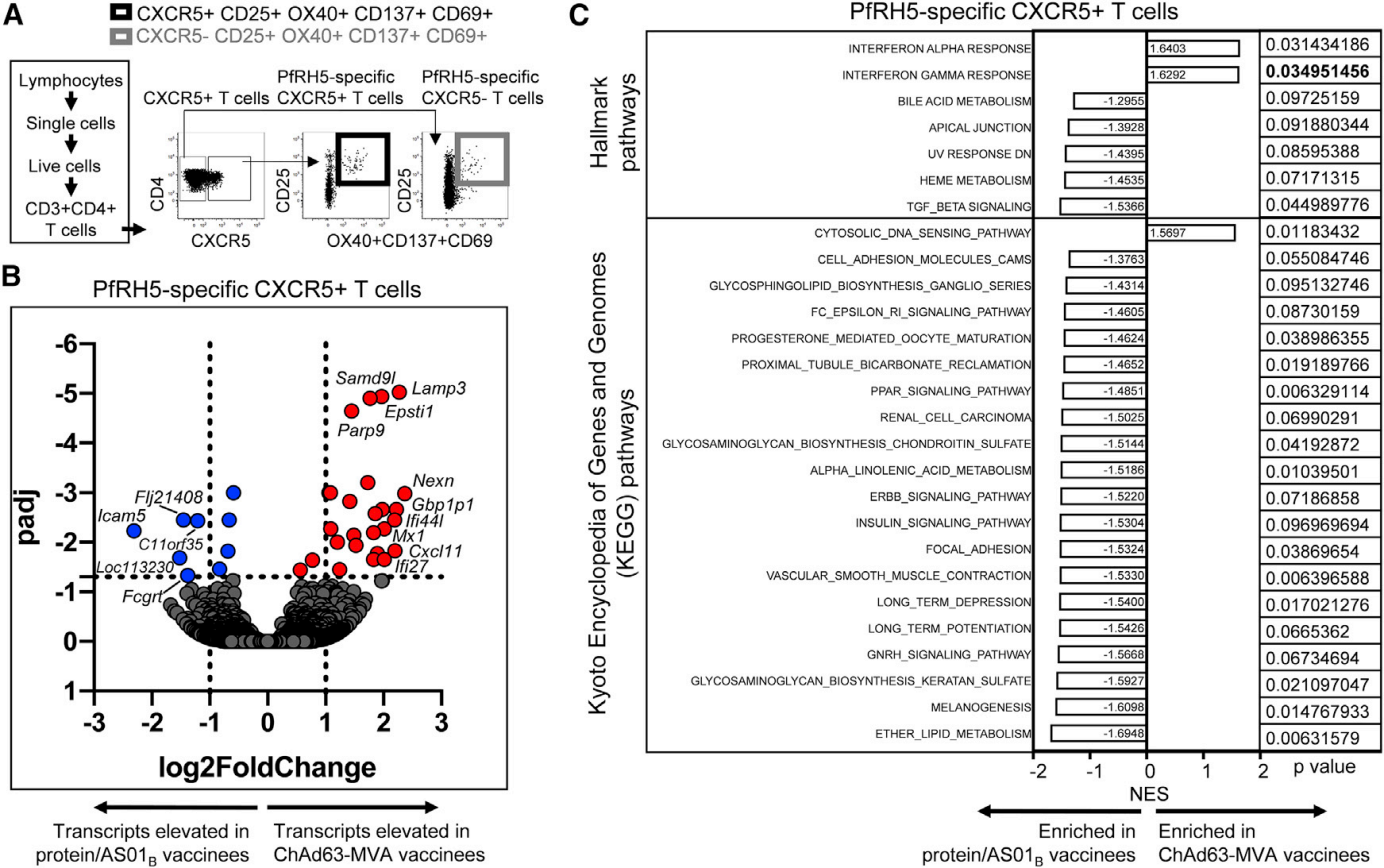
Transcriptomic analysis of PfRH5-specific CXCR5^+^ cells identifies signatures associated with the superior humoral immune response in protein/AS01_B_ vaccinees PBMCs from 4 weeks following final vaccination from vaccinees receiving PfRH5 delivered using ChAd63-MVA (n = 6) or protein/AS01_B_ (n = 5) platforms were stimulated with a PfRH5 peptide pool (2.5 μL/mL) for 24 h and stained for phenotypic and activation markers. (A) Representative gating strategy for sorting of PfRH5-specific CXCR5^+^ and CXCR5^−^CD4^+^ T cells. (B) Volcano plot illustrating genes that were differentially expressed in PfRH5-specific CXCR5^+^CD4^+^ T cells from ChAd63-MVA versus protein/AS01_B_ vaccinees. FDR-adjusted p values are shown on the y axis and log2 fold change on the x axis. Individual transcript names are shown for some points. (C) Results from Gene Ontology enrichment analysis using Hallmark and Kyoto Encyclopedia of Genes and Genomes (KEGG) databases. The network enrichment score (NES) for each pathway enriched or trending to be enriched (p < 0.1) in PfRH5-specific CXCR5^+^CD4^+^ T cells from either ChAd63-MVA or protein/AS01_B_ vaccinees is illustrated, together with the p value; p values in bold remained significant after FDR correction (q < 0.05).

To provide further insight into qualitative differences in the antigen-specific CD4^+^ T cell responses elicited by the 2 different vaccine platforms, RNA sequencing (RNA-seq) was performed on PfRH5-specific CXCR5^+^ (cTfh) and CXCR5^−^ (non-Tfh) CD4^+^ T cells sorted from samples from 6 ChAd63-MVA vaccinees and 5 protein/AS01_B_ vaccinees (4 weeks following final vaccination). PfRH5-specific cells were identified on the basis of activation marker upregulation following stimulation with the PfRH5 peptide pool (Figure 4A).

Following false discovery rate (FDR) adjustment, 26 genes were shown to be expressed at significantly lower and 9 at significantly higher levels in the protein/AS01_B_ vaccinees' PfRH5-specific cTfh cells (Table 2). The genes significantly elevated in ChAd63-MVA vaccinees included multiple genes encoding proteins that regulate the cellular response to, or are upregulated by, IFNs (e.g., *PARP9*, *PARP14*, *EPSTI1*, *IFI44L*, *CXCL11*, *IFI27*, *IFI44*, *GBP4*, and *MX1*)*,* together with some indicators of T cell activation (e.g., *CD38*, *LAMP3*; Table 2; Figure 4B). Further exploration of the transcriptomic signatures of PfRH5-specific CXCR5^+^ cells by gene set enrichment analysis (GSEA) indicated significant enrichment (p < 0.05, q < 0.05) of the IFN-γ response pathway in PfRH5-specific cTfh cells from ChAd63-MVA vaccinees, together with a trend for the enrichment of IFN-α and cytosolic viral DNA-sensing pathways (Figure 4C). Conversely, pathways showing a trend for enrichment in PfRH5-specific cTfh cells from protein/AS01_B_ vaccinees included the peroxisome proliferator-activated receptor (PPAR), fragment crystallizable epsilon receptor 1 (FcεRI), and transforming growth factor β (TGF-β) signaling pathways. TGF-β is among the cytokines that promote the differentiation of human naive T cells into Tfh cells after antigenic stimulation,^38^^,^^39^ while the FcεRI and PPAR signaling pathways are involved in driving Th2 differentiation and the production of IL-4, IL-5, and IL-13 by CD4^+^ T cells (Th2 cytokines that induce B cell activation and differentiation, promoting antibody production^40^, ^41^, ^42^, ^43^). These findings suggest a possible mechanistic basis for the stronger antigen-specific cTfh-inducing capacity of the protein/AS01_B_ vaccine platform, and furthermore support our conclusion that the functional Th2 bias in the overall antigen-specific CD4^+^ T cell response elicited in protein/AS01_B_ vaccinees (Figure 2) is also reflected within the antigen-specific cTfh cell population (Figure 3).

**Table 2:** Genes exhibiting significant differential expression in PfRH5-specific CXCR5^+^CD4^+^ T cells from ChAd63-MVA versus protein/AS01_B_ vaccinees

Gene name	log2 fold change^a^	p^b^	padj^c^
**Genes significantly elevated in PfRH5-specific CXCR5**^**+**^**cells from ChAd63-MVA versus protein/AS01**_**B**_**vaccinees (padj < 0.05)**			

LAMP3	2.272789216	6.58E−10	9.52E−6
EPSTI1	1.965551847	1.60E−9	1.16E−5
SAMD9L	1.767361405	2.59E−9	1.25E−5
PARP9	1.445906493	6.26E−9	2.26E−5
GBP1	1.728592469	2.16E−7	6.25E−4
PARP14	1.08386039	5.00E−7	9.97E−4
MT2A	1.055914157	5.51E−7	9.97E−4
NEXN	2.367357699	6.47E−7	1.04E−3
GBP4	1.416953658	1.04E−6	1.50E−3
GBP1P1	2.222556141	1.71E−6	2.20E−3
XAF1	1.979815403	1.82E−6	2.20E−3
IFI44	1.859295207	2.38E−6	2.65E−3
IFI44L	2.192709426	3.77E−6	3.54E−3
PARP12	1.089549049	7.07E−6	5.39E−3
MX1	2.011280932	7.07E−6	5.39E−3
CD38	1.827598259	9.29E−6	6.40E−3
OAS2	1.488737846	1.10E−5	7.24E−3
IFIH1	1.204575617	1.59E−5	9.98E−3
ETV7	1.520984294	1.91E−5	1.15E−2
CXCL11	2.197707279	2.57E−5	1.48E−2
BCL2L14	1.893427738	3.12E−5	1.67E−2
IFI27	2.013182177	4.51E−5	2.22E−2
IFI6	1.827966249	4.60E−5	2.22E−2
GPATCH1	0.77259541	4.93E−5	2.30E−2
CPNE8	1.242453104	8.09E−5	3.55E−2
PARP11	0.560307668	8.54E−5	3.64E−2

**Genes significantly reduced in PfRH5-specific CXCR5**^**+**^**cells from ChAd63-MVA versus protein/AS01**_**B**_**vaccinees (padj < 0.05)**			

TPGS2	−0.591165012	4.24E−7	9.97E−4
FLJ21408	−1.456625333	3.46E−6	3.54E−3
TPP1	−0.665841538	3.92E−6	3.54E−3
FCGRT	−1.211341921	4.34E−6	3.70E−3
ICAM5	−2.31437469	8.14E−6	5.89E−3
ADAM8	−0.685269989	2.71E−5	1.51E−2
LOC113230	−1.523476907	3.99E−5	2.06E−2
CRTAP	−0.834799548	7.69E−5	3.48E−2
C11orf35	−1.380148661	1.12E−4	4.65E−2

FDR, false discovery rate.

a Elevation or reduction in ChAd63-MVA versus protein/AS01_B_ vaccinees.

b Difference between groups, unadjusted p value.

c False discovery rate (FDR) adjp value. Only genes for which padj < 0.05 are listed.

In the larger, more heterogeneous PfRH5-specific non-Tfh CD4^+^ T cell population, genes expressed at significantly higher levels in ChAd63-MVA vaccinees included *TBX21,* which encodes the Th1 master transcription factor Tbet, as well as genes indicative of greater T cell activation and survival potential (*CD33*, *IL-15*, *LAMP3*, *JUND*) and a response to IFN (*CXCL10*, *IFI27*) (Figure S7; Table S1). GSEA suggested a trend toward enrichment of genes in IFN-triggered pathways following ChAd63-MVA, versus a trend for the upregulation of the mammalian target of rapamycin (mTOR) signaling pathway and numerous pathways involved in amino acid metabolism after protein/AS01_B_ (Figure S7). mTOR promotes aerobic glycolysis and plays a crucial role in effector Th1/Th2/Th17 cell differentiation, expansion, and effector function^44^; these results therefore suggest the induction of antigen-specific CD4^+^ T cells with potent Th1/Th2/Th17 effector capacity in protein/AS01_B_ vaccinees, consistent with high levels of effector cytokine release following PfRH5 stimulation of PBMCs from this group (Figures 2 and S5). These findings further underscore the impact of the vaccine platform on the quality of the antigen-specific effector CD4^+^ T cell response elicited in vaccine recipients.

### Frequencies of antigen-specific cTfh cells correlate with peak anti-PfRH5 IgG concentration, frequency of PfRH5-specific mBCs, and purified IgG *in vitro* neutralization activity

**Figure 5:**
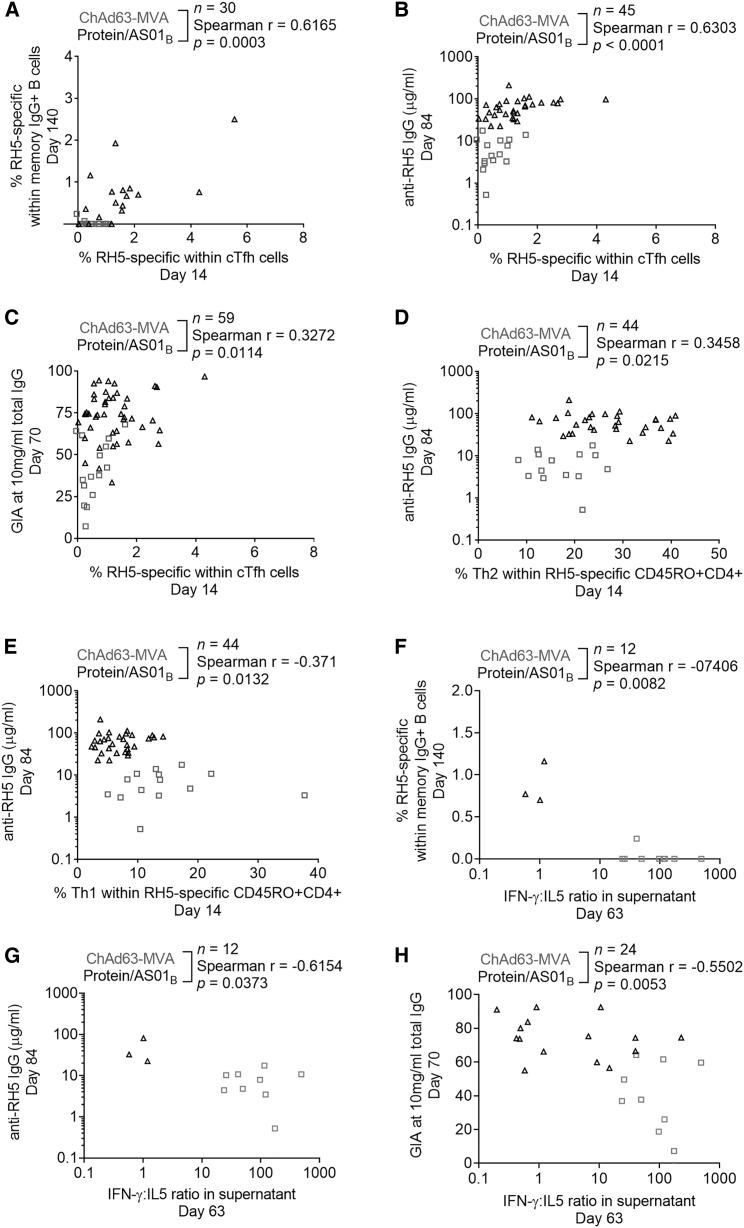
cTfh cell responses and parameters of Th1:Th2 skew correlate with key markers of humoral immunogenicity (A–C) Spearman correlation analyses were performed to interrogate the relationships between the frequency of PfRH5-specific cTfh cells at day 14 and the frequency of IgG^+^ mBCs that were PfRH5 specific at day 140 (A), the serum anti-PfRH5 IgG concentration at day 84 (B), and the purified IgG GIA at 10 mg/mL at day 70 (C). (D–H) Spearman correlation analyses were also performed between the frequency of Th2 (CXCR3^−^CCR6^−^) (D) or Th1 (CXCR3^+^CCR6^−^) (E) cells within PfRH5-specific CD45RO^+^CD4^+^ T cells at day 14 and serum anti-PfRH5 IgG concentration at day 84, as well as between the IFN-γ:IL-5 ratio in day 63 supernatants and the frequency of IgG^+^ mBCs that were PfRH5 specific at day 140 (F), serum anti-PfRH5 IgG concentration at day 84 (G), and the purified IgG GIA at 10 mg/mL at day 70 (H). Sample sizes, Spearman r, and p values annotated on the graphs refer to analyses of pooled samples from both vaccine platforms. In all of the panels, each point represents a vaccinee.

Our interest in understanding the antigen-specific cTfh cell response in the ChAd63-MVA and protein/AS01_B_ platforms is based on the hypothesis that Tfh cell activation is required for induction of a strong humoral response. We therefore used Spearman correlation analyses to assess the relationship between PfRH5-specific cTfh cell frequencies and 3 major readouts of humoral immunogenicity: the frequency of IgG antibody-secreting cells (ASCs) that are PfRH5 specific (day 63), peak anti-PfRH5 serum IgG concentration (day 84), and frequency of PfRH5-specific cells within the IgG^+^ mBC population (day 140; gating strategy shown in Figure S8). We additionally performed correlation analyses with an *in vitro* functional antibody parameter: neutralizing activity of purified total IgG (day 70) in a GIA assay against blood-stage *P. falciparum* parasites, a readout which we have demonstrated to constitute a mechanistic correlate of protection.^30^^,^^31^ Detailed analysis of these (and other) humoral immunogenicity parameters in each vaccine trial is or will be reported in the primary trial publications^1^ (A.M.M. et al., data not shown). These 4 parameters each correlated with days 14 and/or 63 cTfh cell frequencies (Figures 5A–5C, S9, and S10; see graphs for n, Spearman r, and p values). Although there was no relationship between the frequency of PfRH5-specific cTfh cells at day 14 and the PfRH5 plasmablast response as measured by ASC ELISpot, we did observe a significant correlation with PfRH5-specific cTfh cell frequencies at day 63 (Figure S9; note ASC ELISpot data available for protein/AS01_B_ vaccinees only). Consistent with this, the day 63 frequency of plasmablasts within the total B cell population (measured by flow cytometry for vaccinees from both trials) also correlated with the frequency of PfRH5-specific cTfh cells at day 63 but not day 14 (Figure S10). Conversely, we observed a significant correlation between the frequency of IgG^+^ PfRH5-specific mBCs at day 140 and antigen-specific cTfh cells at day 14 (Figure 5A), but not day 63 (Figure S9), although this was putatively due to a reduction in statistical power resulting from a smaller day 63 sample size. Finally, we observed significant correlations between both days 14 and 63 antigen-specific cTfh cell frequencies and peak anti-PfRH5 IgG (Figures 5B and S9) and GIA (Figures 5C and S9). Notably, the correlations between PfRH5-specific cTfh cell responses and anti-PfRH5 IgG concentration or PfRH5-specific mBC frequencies persisted when protein/AS01_B_ vaccinees were analyzed alone (data not shown).

### Th2 qualitative skew associated with protein/AS01_B_ platform may be linked to superior IgG B cell responses

In light of published data suggesting that Tfh1 cells are inferior helpers of B cell humoral responses (reviewed in Koutsakos et al.^15^) and our own observations demonstrating a difference in Th1:Th2 skew between the ChAd63-MVA and protein/AS01_B_ platforms, we explored whether there was any relationship between humoral immunity parameters and indicators of Th/Tfh cell quality. For the latter, we focused on those readouts for which we had previously observed statistically significant differences between the vaccine platforms: frequencies of Th1- or Th2-phenotype cells within PfRH5-specific CD4^+^ T cells at day 14; frequency of Tfh2-phenotype cells within total PfRH5-specific cTfh cells at day 14; and the Th1:Th2 cytokine ratio (IFN-γ:IL-5) at day 63 (summarized in Figures 5D–5H and Table S2). A weak positive correlation was observed between the PfRH5-specific mBC response and the PfRH5-specific Tfh2 cell frequency (Table S2), while the PfRH5-specific Th2 cell frequency correlated with anti-PfRH5 serum IgG (Figure 5D) and GIA (Table S2). Conversely, the frequency of Th1 cells within the PfRH5-specific CD4^+^ T cell population correlated negatively with day 84 anti-PfRH5 IgG (Figure 5E) and day 140 anti-PfRH5 IgG^+^ mBC frequency (Table S2). Consistent with an association between a Th2/Tfh2 CD4^+^ T cell skew and a superior IgG^+^ B cell response, the IFN-γ:IL-5 ratio in day 63 supernatants also negatively correlated with day 140 anti-PfRH5 IgG^+^ mBC frequency, day 84 anti-PfRH5 serum IgG, and day 70 GIA (Figures 5F–5H). While both platforms induce polyfunctional responses, our data suggest a link between a qualitative shift toward a more Th2-biased CD4^+^ Th cell and cTfh cell response and the enhanced humoral immunogenicity of the protein/AS01_B_ platform relative to ChAd63-MVA.

## Discussion

In this study, we directly compared antigen-specific cTfh cell responses elicited by 2 different vaccine platforms in humans: heterologous viral vectors (e.g., similar to those deployed for Ebola) and protein with AS01_B_ adjuvant (part of GlaxoSmithKline’s adjuvant systems; as used in the RTS,S malaria vaccine currently in pilot implementation and the highly successful shingles vaccine Shingrix). We chose to compare the responses elicited in trials in which each platform was deployed in the regimen typically used clinically: a 2-dose heterologous vector prime-boost regimen for ChAd63-MVA and a 3-dose regimen for the protein/AS01_B_. Historical pre-clinical and clinical data have determined that the use of >2 doses in a viral vector regimen does not further improve antibody responses,^45^^,^^46^ while conversely, at least 3 doses are routinely required for subunit vaccines.^47^ Both platforms have shown promise against different pathogens, but there has yet to be a nuanced comparison of the human CD4^+^ Tfh cell responses they elicit and correlation with humoral immunogenicity. We demonstrated that protein/AS01_B_ drove a more robust PfRH5-specific cTfh cell response than the ChAd63-MVA heterologous viral vector platform and that antigen-specific cTfh frequencies correlated with key markers of humoral immunity, including antigen-specific IgG concentration and mBC frequency. Qualitatively, we observed that although Tfh17-phenotype cells predominated within the antigen-specific cTfh population elicited in both vaccine trials, the protein/AS01_B_ platform induced a higher proportion of Tfh2 cells within the antigen-specific cTfh cell population, while the ChAd63-MVA vaccinees conversely had a higher frequency of Tfh1 cells. These data were strongly supported by RNA-seq data from sorted PfRH5-specific cells and measurements of Th cytokines in supernatants from PfRH5 peptide-stimulated PBMCs. Importantly, however, these correlations are not simply artefacts of the protein/AS01_B_ platform being more immunogenic. IFN-γ ELISpot data clearly showed that the ChAd63-MVA platform is a more robust driver of T cell IFN-γ responses; thus, readouts indicating superior immunogenicity of the protein/AS01_B_ platform appear to be those specifically linked to the humoral response. The PfRH5-specific cTfh cell RNA-seq analysis detected a higher expression of several genes related to IFN signaling in ChAd63-MVA vaccinees, while multiple genes related to Tfh and Th2 cell differentiation were more strongly expressed in protein/AS01_B_ vaccinees (e.g., PPAR, FcεRI, TGF-β). Our data support a model in which the protein/AS01_B_ platform drives a greater Th2/Tfh2 bias in the antigen-specific CD4^+^ response relative to the ChAd63-MVA platform (although the Th2/Tfh2 component does not dominate the overall response in protein/AS01_B_ vaccinees; our analyses indicate that the majority of antigen-specific Tfh elicited by both platforms are Tfh17 cells).

In-depth analysis of GC Tfh responses at serial post-vaccination time points in clinical trials is hampered by the difficulty in sampling lymphoid tissues in humans. However, cTfh cells in the peripheral blood memory CD4^+^ T cell pool exhibit phenotypic, functional, gene expression, and T cell receptor repertoire profiles similar to those of lymphoid tissue CD4^+^ Tfh populations.^8^, ^9^, ^10^, ^11^, ^12^, ^13^, ^14^ Although the relationship between these cTfh cells and lymphoid tissue Tfh subsets remains incompletely understood,^48^ it is increasingly accepted that analysis of cTfh populations can provide insight into post-vaccination GC responses.^16^^,^^18^^,^^20^^,^^22^^,^^24^, ^25^, ^26^^,^^28^ Plasma concentrations of CXCL13 have also been reported as a proxy peripheral indicator of GC activity.^49^^,^^50^ However, we did not observe any consistent post-vaccination trends in plasma CXCL13 levels (data not shown), suggesting that this is not a robust universal indicator of GC responses. Others have similarly failed to observe post-vaccination changes in CXCL13 concentration,^23^ and the original report, in fact, observed this only with HIV and yellow fever vaccines, but not with an inactivated trivalent influenza vaccine.^49^ In the absence of a reliable chemokine (or other) biomarker, the value of insight from antigen-specific cTfh cell frequencies into ongoing responses in GCs is even greater.

Induction of a superior humoral response by the protein/AS01_B_ platform was associated with not only a higher-magnitude but also a more Th2-biased antigen-specific cTfh response than that elicited by ChAd63-MVA immunization. This observation is consistent with prior studies showing that bulk memory cTfh2 and cTfh17 cells produce high levels of IL-21 and provide help to both naive B cells and mBCs *in vitro*, whereas cTfh1 cells support mBC responses poorly and do not provide help to naive B cells.^8^^,^^9^^,^^11^ Likewise, seasonal influenza vaccines elicit an antigen-specific cTfh1 response;^16^^,^^18^^,^^25^^,^^28^ and whereas the cTfh1 cells activated following vaccination are able to provide help for antigen-loaded influenza-specific mBCs *in vitro*, they produce insufficient IL-21 to support naive B cell activation.^16^ Furthermore, although seasonal influenza vaccines elicit an initial increase in serum antibody avidity, this is not sustained, suggesting that although influenza-specific cTfh1 may drive high-affinity preexisting mBCs to undergo activation and plasmablast differentiation, they may provide inefficient support for sustained GC responses.^51^

Our data also cohere to the literature within the malaria field on the role of Tfh cell subsets in anti-malarial B cell responses. Sorted Tfh2 or Tfh17 cells from malaria-exposed Malian children are capable of inducing B cells to proliferate and produce IgG in co-culture assays,^52^ but malaria infection induces Th1 cytokines that drive the inferior Tfh1 subset.^52^ More recently, the co-administration of ChAd63 and MVA viral vectors with RTS,S led to an increase in CXCR3^+^ cTfh cells, which negatively correlated with IgG responses to the RTS,S antigen circumsporozoite protein.^27^ Both articles suggested that Tfh1 cell responses, and the associated pro-inflammatory IFN-γ signaling, are detrimental for inducing humoral responses against malarial antigens (also reviewed in Hansen et al.^53^). It therefore follows that for a pathogen in which protection is antibody mediated, such as blood-stage malaria, the optimal response is one that promotes antigen-specific Tfh2/Th17 responses rather than Tfh1.^53^ We found that the ratio of IFN-γ:IL-5 (i.e., Th1:Th2) cytokines in supernatants from PfRH5-stimulated PBMCs negatively correlates with both the frequency of PfRH5-specific IgG^+^ mBCs and GIA (p = 0.0082 and p = 0.0053, respectively; Table S2). Unfortunately, targeting a Tfh2/Th17 response may be particularly challenging in the context of endemic malaria, in which immune responses may be chronically skewed toward a pro-inflammatory phenotype.

Of relevance is a recent publication reporting cTfh cell activation in Tanzanian volunteers (a malaria-exposed population) following administration of the blood-stage vaccine candidate P27A with either an oil-in-water emulsion (GLA-SE) or an alum adjuvant.^13^ Here, a more robust expansion of bulk cTfh cells in the GLA-SE arm indicated that altering adjuvant is a feasible approach to target enhanced Tfh cell help for B cell responses. While the authors reported no differences in the quality of the cTfh cell response between the 2 adjuvant groups, it is possible that this was related to the lack of an antigen-specific approach and/or the small sample size (n = 8 per group). Alternatively, it may be that differences in innate responses to the P27A peptide with GLA-SE versus alum are less pronounced than the differences in responses driven by ChAd63-MVA as compared to protein/AS01_B_, or that a putative intrinsic Th1 bias in malaria-exposed individuals is strong enough to obscure any subtle changes detectable in malaria-naive UK populations. It will be of great interest to compare antigen-specific Tfh responses in (PfRH5) vaccine trials in malaria-exposed vaccinees to ascertain whether a protein/adjuvant platform is sufficient to induce a Th2 bias as compared to ChAd63-MVA, equivalent to what we have observed in malaria-naive vaccinees in the United Kingdom.

*In vitro* GIA and *in vivo* growth inhibition data from malaria challenge trials have convincingly demonstrated that protection from blood-stage malaria can be achieved with a PfRH5 vaccine, contingent on the induction of sufficiently high titers of anti-RH5 IgG^30^^,^^31^ (A.M.M. et al., data not shown). Understanding the mechanisms by which this IgG response can be modulated is therefore critical. Our results are informative for guiding the next stages of blood-stage malaria vaccine development, and vaccine development against any pathogen in which protection is antibody mediated and a sustained high-titer antibody response is required for prophylaxis. Of note, the associations that we have observed between day 14 cTfh cell responses and IgG and mBC responses as late as day 140 suggest that relatively early cTfh cell responses may have predictive values of peak humoral immunogenicity and longevity.

Our data strongly indicate that the vaccine platform affects the magnitude and quality of post-vaccination antigen-specific CD4^+^ Th cell and cTfh cell responses. Further investigations of PfRH5 vaccine candidates or other antigens could focus on interrogating the links between adjuvant selection and the induction of optimal Tfh cell help, as well as making not only quantitative but also qualitative comparisons between the cTfh responses induced by protein/AS01_B_ and newer platforms such as novel virus-like particle (VLP) constructs or nucleoside-modified mRNA-containing lipid nanoparticles.^54^ Interrogation of the impact of antigen dose, which was outside the scope of this study, may also be warranted. Ultimately, an improved understanding of the cellular mediators of humoral responses will help guide vaccine development against pathogens where durable, high concentration antibody is required to mediate protection.

### Limitations of study

The main limitation of this study is the relatively small sample size, particularly for some of the correlations. This was predominantly due to restrictions in PBMC availability and sample exclusions in the flow cytometry analyses when the parent population contained <50 cells (as detailed in the STAR methods and the figure legends). Our conclusions could therefore be strengthened by repeating the vaccine platform comparison with samples from additional clinical trials, perhaps with a different antigen or in a different population. Similarly, larger sample sizes would increase the statistical power of the analyses and thus improve the capacity to detect smaller differences between platforms. A larger sample size would have also permitted an interrogation of any impact of dosing within each clinical trial. Finally, while the use of cTfh cells from peripheral blood samples as proxies for the GC Tfh cell response is widely accepted, it would be of great interest to conduct equivalent analyses with draining lymph node aspirate samples.

## STAR★Methods

### Key Resources Table

**Table:** 

REAGENT or RESOURCE	SOURCE	IDENTIFIER
**Antibodies**		

Rat anti-human CXCR5-BB515. Clone RF8B2.	BD	Cat# 564624, RRID:AB_2738871
Mouse anti-human CXCR3-PE-Cy5. Clone 1C6/CXCR3.	BD	Cat# 551128, RRID:AB_394061
Mouse anti-human CD25-PE-Cy7. Clone 2A3.	BD	Cat# 335824, RRID:AB_2868687
Mouse anti-human CD3-PE-TR. Clone 7D6.	Life Tech.	Cat# MHCD0317, RRID:AB_10376002
Mouse anti-human CCR6-PE. Clone 11A9.	BD	Cat# 551773, RRID:AB_394247
Mouse anti-human CD4-APC-H7. Clone SK3.	BD	Cat# 641398, RRID:AB_1645732
Mouse anti-human CD8a-AF700. Clone RPA-T8.	Biolegend	Cat# 301028, RRID:AB_493745
Mouse anti-human CD38-BV785. Clone HIT2.	Biolegend	Cat# 303530, RRID:AB_2565893
Mouse anti-human CD45RA-BV711. Clone HI100.	Biolegend	Cat# 304138, RRID:AB_2563815
Mouse anti-human CCR7-BV650. Clone G043H7.	Biolegend	Cat# 353234, RRID:AB_2563867
Mouse anti-human ICOS-Biotin. Clone ISA-3.	Invitrogen	Cat# 13-9948-82, RRID:AB_467004
Mouse anti-human PD1-BV421. Clone EH12.2H7.	Biolegend	Cat# 329920, RRID:AB_10960742
Mouse anti-human Ki67-PerCP-ef710. Clone 20Raj1.	Invitrogen	Cat# 46-5699-42, RRID:AB_10804653
Rat anti-human Foxp3-APC. Clone PCH101.	eBioscience	Cat# 17-4776-42, RRID:AB_1603280
Mouse anti-human CD183-APC. Clone 1C6/CXCR3.	BD	Cat# 550967, RRID:AB_398481
Rat anti-human CXCR5 APC-R700. Clone RF8B2.	BD	Cat# 565191, RRID:AB_2739103
Mouse anti-human CCR6-BV711. Clone G034E3.	Biolegend	Cat# 353436, RRID:AB_2629608
Mouse anti-human CD14- BV510. Clone M5E2.	Biolegend	Cat# 301842, RRID:AB_2561946
Mouse anti-human CD19-BV510. Clone SJ25C1.	BD	Cat# 562947, RRID:AB_2737912
Mouse anti-human CD137-BV650. Clone 4B4-1.	Biolegend	Cat# 309828, RRID:AB_2572193
Mouse anti-human CD45RO-BV785. Clone UCHL1.	Biolegend	Cat# 304234, RRID:AB_2563819
Mouse anti-human OX40-PE. Clone L106.	BD	Cat# 340420, RRID:AB_400027
Mouse anti-human CD69-PE-Cy5. Clone FN50.	BD	Cat# 555532, RRID:AB_395917
Mouse anti-human CD8-PE-TR. Clone 3B5.	Invitrogen	Cat# MHCD0817, RRID:AB_10372359
Mouse anti-human CD3-BV605. Clone UCHT1.	Biolegend	Cat# 300460, RRID:AB_2564380
Mouse anti-human CD19-PE-Cy7. Clone SJ25C1.	BD	Cat# 557835, RRID:AB_396893
Mouse anti-human IgG-BB515. Clone G18-145.	BD	Cat# 564581, RRID:AB_2738854
Mouse anti-human CD27-BV711. Clone M-T271.	BD	Cat# 564893, RRID:AB_2739003
Mouse anti-human CD21-BV421. Clone B-ly4.	BD	Cat# 562966, RRID:AB_2737921
Mouse anti-human IgM-BV510. Clone G20-127.	BD	Cat# 563113, RRID:AB_2738010
Mouse anti-human CD24-APC-H7. Clone ML5.	Biolegend	Cat# 311132, RRID:AB_2566347
Mouse anti-human CD19-BV650. Clone HIB19.	Biolegend	Cat# 302238, RRID:AB_2562097

**Biological samples**		

Pooled human AB plasma	Sigma	H4522

**Chemicals, peptides, and recombinant proteins**		

Live/Dead Aqua	Invitrogen	L34966
Streptavidin-BV605	BD	Cat# 563260, RRID:AB_2869476
Streptavidin-BB515	BD	Cat# 564453, RRID:AB_2869580
Fixable viability stain 780	BD	Cat# 565388, RRID:AB_2869673
Streptavidin-PE	Invitrogen	S866
Streptavidin-APC	eBioscience	405207
Monobiotinylated RH5	This paper	N/A
Benzonase endonuclease	Merck	70746-3
Trustain	Biolegend	Cat# 422302, RRID:AB_2818986
50 20-mer PfRH5 peptides	Synthesized by NeoScientific, obtained from Simon Draper	n/a

**Critical commercial assays**		

Foxp3/Transcription Factor Staining Buffer Set	eBioscience	00-5523-00
Human Pan B Cell Enrichment Kit	StemCell	19554
Cytofix/Cytoperm	BD	Cat# 554714, RRID:AB_2869008
LEGENDplex Human Th Cytokine Panel Kit	Biolegend	Cat# 740001 and 740722, RRID:AB_2784515
RNeasy mini kit	QIAGEN	74104
Clontech Ultra Low Input v4 kit	Illumina	FC-131-1096

**Deposited data**		

RNaseq from RH5-specific CXCR5+ and CXCR5- CD4+ T cells	This paper	NCBI SRA database, BioProject accession number PRJNA602552

**Software and algorithms**		

BD FACSDiva v8	BD	n/a
FlowJo v9-10	TreeStar	n/a
Prism v8	GraphPad	n/a
LEGENDplex Data Analysis Software for LEGENDplex Multi-analyte Flow Assay Kits v8	Biolegend	n/a
STAR v 2.3.1 s	n/a	n/a
DESeq2 v1.0.17	n/a	n/a
GSEA v3 and MsigDB v6 containing Hallmark genesets and Kegg curated pathways (CP)	n/a	n/a

### Resource availability

#### Lead contact

Further information and requests for resources and reagents should be directed to and will be fulfilled by the Lead Contact, Carolyn Nielsen (carolyn.nielsen@ndm.ox.ac.uk).

#### Materials availability

This study did not generate new unique reagents.

#### Data and code availability

The RNASeq data generated during this study are available from the NCBI SRA database, BioProject accession number PRJNA602552: https://www.ncbi.nlm.nih.gov/Traces/study/?acc=PRJNA602552&o=acc_s%3Aa.

### Experimental model and subject details

This study entailed comparison of the immune responses elicited in two clinical trials employing different vaccine platforms to deliver the same antigen (PfRH5): heterologous viral vectors consisting of a ChAd63-RH5 prime, followed by a MVA-RH5 boost^1^ (ClinicalTrials.gov Identifier: NCT02181088), and a three-dose series of full length PfRH5 protein (RH5.1) with AS01_B_ adjuvant (ClinicalTrials.gov Identifier: NCT02927145^2^; adjuvant provided by GlaxoSmithKline). The PfRH5 sequence, encoded as a transgene in ChAd63-RH5 and MVA-RH5 or expressed from a *Drosophila* Schneider 2 (S2; ExpreS2ion Biotechnologies) stable cell line for the protein/AS01_B_ platform, is based on the 3D7 clone of *P. falciparum*. This sequence was conserved between the two trials, with the exception of six threonine to alanine substitutions in the full-length protein construct, RH5.1, to prevent N-linked glycosylation during protein production and the addition of ‘C-tag’ to allow protein purification.^2^

The ChAd63-MVA and protein/AS01_B_ clinical trials from which samples were used in this study were approved by the Oxford Research Ethics Committee A in the UK (REC references 14/SC/0120 and 16/SC/0345, respectively) as well as by the UK Medicines and Healthcare products Regulatory Agency (MHRA; references 21584/0331/001-0001 and 21584/0362/001-0001, respectively). All volunteers gave written informed consent.

Vaccine regimens are presented in Table 1. A comparison of responses between standard regimen groups and the delayed fractional dose regimen group within the protein/AS01_B_ trial was outside the scope of this study; samples from time points after the delayed fractional dose were not included in the analyses presented here, with the exception of one sample employed for the transcriptome work. As there were no significant differences in the serum anti-PfRH5 IgG concentration or the PfRH5-specific T cell responses detected in IFN-γ ELISPOT assays between dose groups within the standard regimens of each trial (Tables 1 and2)^1^ (data not shown), groups were pooled for all analyses performed in this study to maximize the statistical power to detect differences between vaccine platforms.

Demographic characteristics of vaccinees are presented in Table 1; note that this table summarizes information for only those vaccinees included in the study presented in this paper (selected based on PBMC availability), rather than all vaccinees within the respective trials. There were no significant differences between trials in the proportion of female vaccinees (Fisher’s exact test, p = 0.2302) or the age of vaccinees (Mann-Whitney test, p = 0.2650). The maximum sample size was n = 15 of the 24 vaccinees in the ChAd63-MVA trial and n = 57 of the 64 vaccinees in the protein/AS01_B_ trial. However, as sufficient PBMCs were not available from every time point to enable analyses to be performed in all 72 vaccinees, group sizes for some assays were lower (as specified in the figure legends). Datasets for certain parameters within the flow cytometry analyses are also smaller than the group sizes reported in the legends as samples were excluded from downstream analysis if the parent cell population contained fewer than 50 events. Similarly, intra-trial comparisons were limited to only those vaccinees where samples were available from all time points.

### Method details

Existing anti-RH5 IgG, IFN-γ ELISPOT, antibody secreting cell (ASC) ELISPOT, and growth inhibitory activity (GIA) datasets reported elsewhere were used in correlation analyses. In brief, for anti-PfRH5 IgG, total anti-PfRH5 IgG AU titers were quantified in pre- and post-vaccination sera samples using a standardized enzyme-linked immunosorbent assay (ELISA^55^) against full-length RH5 protein (RH5.1). The reciprocal of the test sample dilution giving an optical density at 405nm (OD_405_) of 1.0 in the standardized assay was used to assign an ELISA unit value of the standard. A standard curve and Gen5 ELISA software (Biotech, UK) was used to convert the OD_405_ of individual test samples into arbitrary units (AU). These responses in AU were then reported in μg/ml concentrations following generation of a conversion factor by calibration-free concentration analysis (CFCA), as previously reported for ChAd63-MVA.^1^ For *ex vivo* IFN-γ ELISPOTs, PBMC were isolated from fresh blood samples, stimulated with a PfRH5 peptide pool at a final concentration of 5μg/ml of each peptide, and antigen-specific IFN-γ-producing cell frequencies were calculated from IFN-γ spot-forming units (SFU) per million PBMC. Background responses in unstimulated control wells were subtracted from peptide-stimulated wells.^1^ Fresh PBMC were also used for *ex vivo* ASC ELISPOTs (protein/AS01_B_ vaccinees only). Here, plates were coated with RH5.1 protein or polyvalent Ig (H1700, Caltag) and PfRH5-specific ASCs were reported as a percentage of total IgG-secreting B cells.^1^^,^^56^ For both ELISPOTs, plates were counted using an AID ELISPOT plate reader. GIA of post-vaccination serum samples at 10mg/ml total IgG was assessed at the GIA Reference Center (NIAID, NIH) as previously described.^1^^,^^57^ In brief, purified IgG samples were incubated with *P. falciparum*-infected red blood cells for 40 hours at 37°C, and biochemical determination of parasite lactate dehydrogenase used to quantify final parasitemia in each well.

#### Flow cytometry assays

Assays detailed individually below. Unless otherwise stated, all samples were acquired on a Fortessa X20 flow cytometer using BD FACSDiva8.0 (both BD Biosciences) and data were analyzed in FlowJo (v10, Treestar). Antibody catalog numbers and clones are summarized in Table S3.

For bulk *ex vivo* T cell and B cell analyses, cryopreserved PBMC were thawed into R10 media (RPMI [R0883, Sigma] supplemented with 10% heat-inactivated FCS [60923, Biosera], 100U/ml penicillin / 0.1mg/mL streptomycin [P0781, Sigma], 2mM L-glutamine [G7513, Sigma]) then washed and rested in IMDM-10 (Iscove’s Modified Dulbecco’s Medium [13390, Sigma] supplemented 10% pooled human AB plasma [H4522, Sigma]) in the presence of benzonase endonuclease (70746-3, Merck). PBMC were incubated with Trustain (422302, Biolegend) to block Fc receptors before staining. For the *ex vivo* T cell assay, prior to intracellular and intranuclear staining cells were fixed and permeabilized with Foxp3 / Transcription Factor Staining Buffer Set (00-5523-00, eBioscience).

For antigen-specific *ex vivo* B cell analysis, PfRH5-specific mBCs were quantified pre- and post-vaccination by staining with fluorophore-conjugated PfRH5 probes. Monobiotinylated PfRH5 was produced by transient co-transfection of HEK293F cells with a plasmid encoding BirA biotin ligase and a plasmid encoding a modified full-length PfRH5. The PfRH5 plasmid was based on ‘RH5-bio’ (gift from Gavin Wright [Addgene plasmid # 47780; http://n2t.net/addgene:47780;RRID:Addgene_47780]^58^) which includes a CD4 tag to improve expression and a biotin acceptor peptide (BAP). RH5-bio was modified prior to transfection to incorporate a ‘C-tag’ for subsequent protein purification, as well as a 15 amino acid deletion at a predicted cleavage site. Monobiotinylated PfRH5 was purified via CaptureSelect affinity C-tag resin (Life Technologies), eluted with 2M MgCl_2_ 20mM Tris pH 7.4, dialyzed in PBS, and stored at −80°C until use. Probes were freshly prepared for each experiment, using a protocol adapted from Franz et al.^59^ and Wang et al.,^60^ by incubation of monobiotinylated PfRH5 with streptavidin-PE or streptavidin-APC at an approximately 4:1 molar ratio to facilitate tetramer generation and subsequently centrifuging to remove aggregates. Cryopreserved PBMC were enriched for B cells with a Human Pan-B cell Enrichment Kit (19554, StemCell), stained, and fixed with Cytofix/Cytoperm (554714, BD Biosciences). Samples were acquired on an LSR II (BD Biosciences) the same day and data were analyzed in FlowJo (version 10, Treestar).

For the *in vitro* activation induced marker (AIM) T cell assay, PBMC obtained from the same cryopreserved vials as in the bulk *ex vivo* assay above were rested for six hours in IMDM-10 (I3390, Sigma) in the presence of benzonase endonuclease (70746-3, Millipore). PBMC were then stimulated for 24 hours at 37°C in IMDM-10 with a PfRH5 peptide pool (20-mer peptides spanning full-length PfRH5 protein, overlapping by 10 amino acids, total of 50 peptides [NeoScientific]^1^) using each peptide at a final concentration of 2.5μg/ml, or with 1μg/ml *Staphylococcal* enterotoxin B (SEB; S-4881, Sigma) as a positive control. Medium only served as a negative control. Anti-human CXCR3-APC was included in the cell culture medium. Following incubation, PBMC were stained and fixed with Cytofix/Cytoperm (554714, BD Biosciences). PfRH5-specific cells were defined using Boolean gating as cells co-expressing CD25 with OX40 and/or CD137 and/or CD69 following stimulation with the PfRH5 peptide pool. The frequency of activated cells in sample-matched unstimulated wells was subtracted to control for non-specific activation. This assay is an adaptation (data not shown) of published AIM methods.^21^^,^^29^^,^^36^^,^^61^

Concentrations of 13 Th cytokines (IL-2, IL-4, IL-5, IL-6, IL-9, IL-10, IL-13, IL-17A, IL-17F, IL-21, IL-22, IFN-γ and TNFα) were measured in supernatants from PfRH5 peptide-stimulated wells in the AIM assay using the LEGENDplex Human Th Cytokine Panel Kit (740001 and 740722, Biolegend), as per the manufacturer’s instructions. Samples were assayed in duplicate and acquired on a LSR II (BD Biosciences). Median fluorescence intensities were used for all analyses, which were performed using the LEGENDplex Data Analysis Software for LEGENDplex Multi-analyte Flow Assay Kits (version 8.0, Biolegend).

#### RNA-seq of PfRH5-specific CXCR5+ and CXCR5- CD4+ T cells

B cell-depleted PBMC (Human Pan-B cell Enrichment Kit [(19554, StemCell]) from cryopreserved samples were available from the four week post-vaccination time point in six ChAd63-MVA vaccinees and six protein/AS01_B_ vaccinees (five vaccinees from the standard regimen and one vaccinee from the delayed fractional dose regimen). These samples were stimulated with a PfRH5 peptide pool as described for the AIM assay. Cells were then stained with a subset of the anti-human antibodies / dyes used for the AIM assay: anti-CXCR5-APC-R700, anti-CD137-BV650, anti-CD4-APC-H7, anti-CD134 (OX40)-PE, anti-CD69-PE-Cy5, anti-CD25-PE-Cy7, anti-CD3-BV605, and LIVE/DEAD AQUA viability dye. PfRH5-specific CXCR5+ and CXCR5- CD4 T cells, identified on the basis of upregulation of CD25 together with CD134, CD137 and/or CD69, were then sorted on a BD FACSAria (BD Biosciences), and total RNA was isolated using a RNeasy mini kit (74104, QIAGEN). The quality of the RNA extracted was checked using an Agilent 4200 Tapestation and resulted in the exclusion of both CXCR5+ and CXCR5- cell RNA samples from one subject in the protein/AS01_B_ vaccine group due to an RIN score > 6.

RNASeq analysis was performed as previously described.^62^ cDNA was amplified using the Clontech Ultra Low Input v4 kit (Takara/Clontech). Sequencing libraries were created using the Nextera library preparation kit (FC-131-1096, Illumina) and quantified by qPCR. Libraries were sequenced using the NextSeq500 platform (Illumina) to a read depth of at least 25 million reads per sample. Reads were aligned to the human genome (*Homo sapiens*/hg19) using STAR, the number of reads mapped to each gene was quantified using HTseq and differential gene expression was performed using DESeq2, including a FDR correction (Benjamini-Hochberg). Pathway analysis was performed using GSEA software from the Broad Institute of MIT and Harvard; briefly, entire expression dataset containing transcript counts was inputted into GSEA database, phenotypes were defined according to vaccine platforms, and Hallmark or Kegg mSigDB were used to define enriched gene sets from previously curated databases.

RNASeq data were deposited to the NCBI SRA database, BioProject accession number PRJNA602552.

### Quantification and statistical analysis

Statistical comparisons within trials utilized Wilcoxon signed rank tests or a Friedman test with Dunn’s correction for multiple comparisons. Comparisons between trials were done with Mann Whitney tests or a Kruskal-Wallis test with Dunn’s correction for multiple comparisons. Associations between parameters were performed with Spearman correlations after pooling samples from both trials. All *p* values (or adjusted *p* values for tests with Dunn’s correction) are two-tailed and are considered significant at the α = 0.05 level. Statistical analyses were performed in Prism (Version 8, GraphPad).

For RNASeq analyses, differences between trials were considered significant after FDR adjustments when *q* < 0.05.

Sample size per assay and statistical tests used are stated in figure legends. Please also see Experimental model and subject details: human studies for further details of sample size and inclusion/ exclusion criteria.

As is common for exploratory immunological analyses, our analysis included many statistical comparisons as we sought to understand the relationship between the many parameters included in our study. We note there are risks inherent with this multiplicity of testing and accept that our conclusions will be strengthened by reproduction of these analyses with different sample sets.

### Additional resources

ChAd63-MVA (NCT02181088): https://clinicaltrials.gov/ct2/show/NCT02181088

Protein/AS01_B_ (NCT02927145): https://clinicaltrials.gov/ct2/show/NCT02927145

## Supplemental information

Document S1. Figures S1–S10 and Tables S1 and S2

Document S2. Article plus supplemental information
