# Supplementary material for: Protein/AS01B vaccination elicits stronger, more Th2-skewed antigen-specific human T follicular helper cell responses than heterologous viral vectors
Source: Cell Rep Med. 2021 Feb 22;2(3):100207. doi: 10.1016/j.xcrm.2021.100207 (PMC7974546; doi:10.1016/j.xcrm.2021.100207)
Supplement: Document S1. Figures S1–S10 and Tables S1 and S2 [file mmc1.pdf]

Cell Reports Medicine, Volume *2*

Supplemental information

Protein/AS01$_B$ vaccination elicits stronger, more Th2-skewed antigen-specific human T follicular helper cell responses than heterologous viral vectors

Carolyn M. Nielsen, Ane Ogbe, Isabela Pedroza-Pacheco, Susanne E. Doeleman, Yue Chen, Sarah E. Silk, Jordan R. Barrett, Sean C. Elias, Kazutoyo Miura, Ababacar Diouf, Martino Bardelli, Rebecca A. Dabbs, Lea Barfod, Carole A. Long, Barton F. Haynes, Ruth O. Payne, Angela M. Minassian, Todd Bradley, Simon J. Draper, and Persephone Borrow

Supplemental Figures and Tables

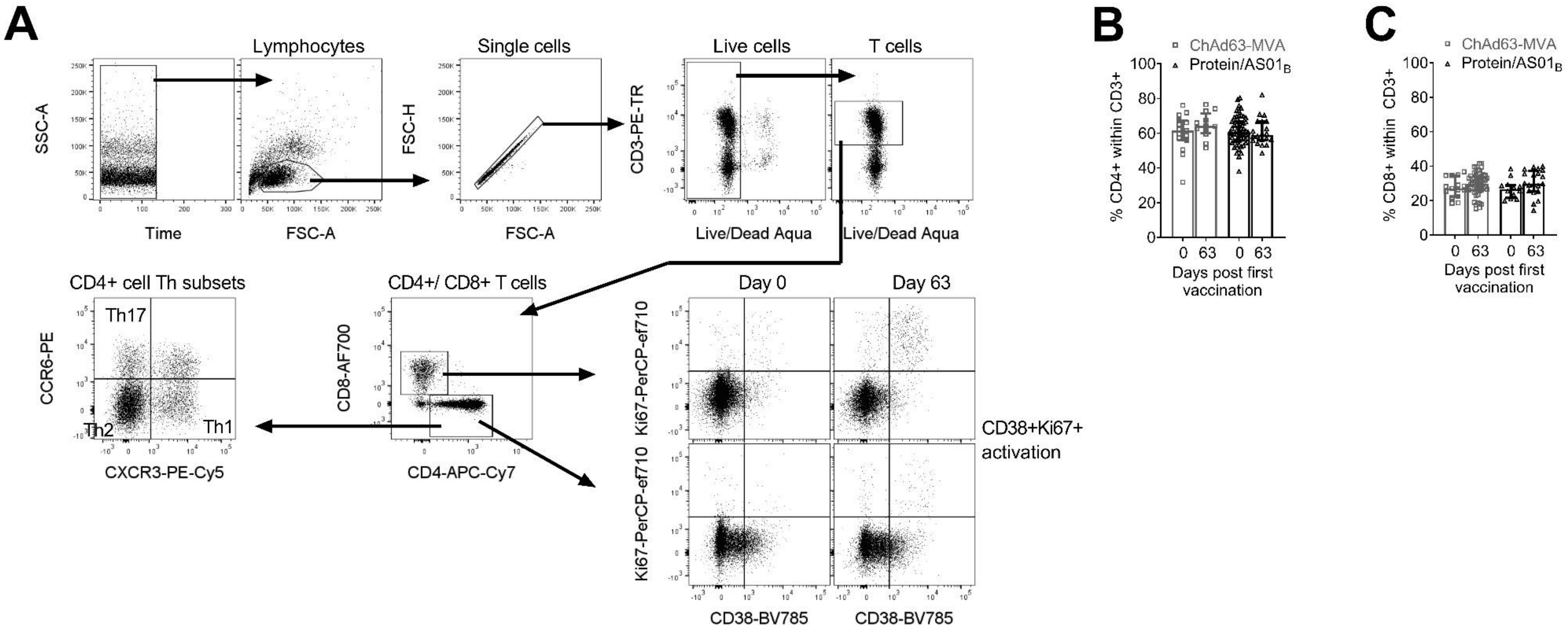

Supplemental Figure 1. Analysis of circulating frequencies of total and activated CD8+ and CD4+ T cells following PfRH5 vaccination with the ChAd63-MVA platform or the protein/AS01$_B$ platform. Related to Figure 1. PBMC from Day 0 and Day 63 (one week following final vaccination) from vaccinees receiving PfRH5 delivered by ChAd63-MVA or protein/AS01$_B$ were stained *ex vivo* for phenotypic and activation markers and analyzed by flow cytometry. **(A)** Gating strategy for definition of CD4+ and CD8+ cells within live, single, CD3+ (T cell) lymphocyte population, stratification of CD4+ T cells into Th1 (CXCR3+CCR6-), Th2 (CXCR3-CCR6-) and Th17 (CXCR3-CCR6+), and expression of activation markers CD38 and Ki67 at Day 0 versus Day 63. Frequencies of total CD4+ T cells **(B)** and CD8+ T cells **(C)** were compared within and between platforms. All available samples are plotted (ChAd63-MVA / protein/AS01$_B$): Day 0, *n*=15/54; Day 63, *n*=12/20. Comparisons were performed with either Wilcoxon tests (within trials) or Mann-Whitney tests (between trials). In all panels, each point represents a vaccinee. Bars and lines denote medians and interquartile ranges, respectively.

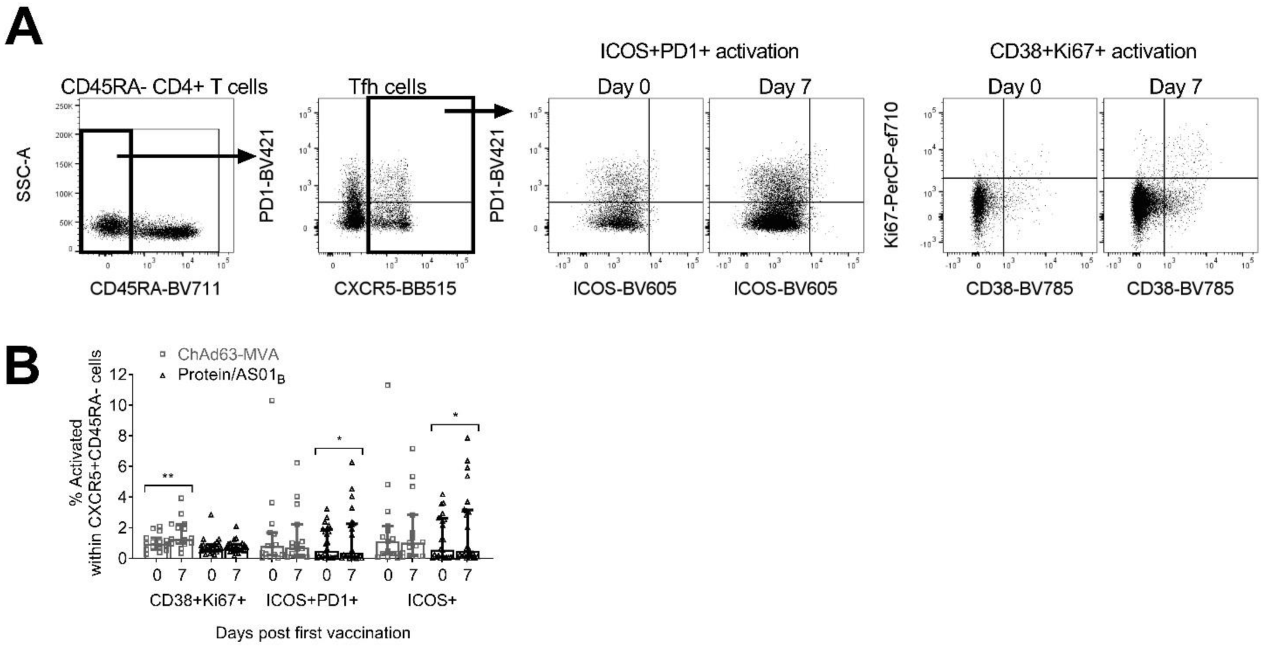

Supplemental Figure 2. Post-vaccination increase in the activation of CXCR5+ CD4+ T cell populations in both ChAd63-MVA and protein/$AS01_B$ vaccinees. Related to Figure 1. PBMC from Day 0, 7, 14, and 63 (one week following final vaccination) from vaccinees receiving PfRH5 delivered by ChAd63-MVA or protein/$AS01_B$ were stained *ex vivo* for phenotypic and activation markers and analyzed by flow cytometry. **(A)** Gating strategy for definition of CD45RA- cells within CD4+ T cells, cTfh cells as CXCR5+ or CXCR5+PD1+ within CD46RA-CD4+ T cells, and expression of activation markers ICOS +/- PD1, or CD38+Ki67+ on CXCR5+ cTfh cells. Total CD4+ T cell gating strategies were as in Supplemental Figure 1. Within CXCR5+ cTfh cells, increases at Day 7 in activated cells that were CD38+Ki67+, ICOS+PD1+, or ICOS+ alone **(B)** were assessed for each platform. All available samples were included (ChAd63-MVA/ protein/$AS01_B$): Day 0, *n*=15/54; Day 7, n=15/24. Comparisons were performed between trials with Mann-Whitney tests. * p<0.05, ** p<0.01. In all panels, each point represents a vaccinee. Bars and lines denote medians and interquartile ranges, respectively.

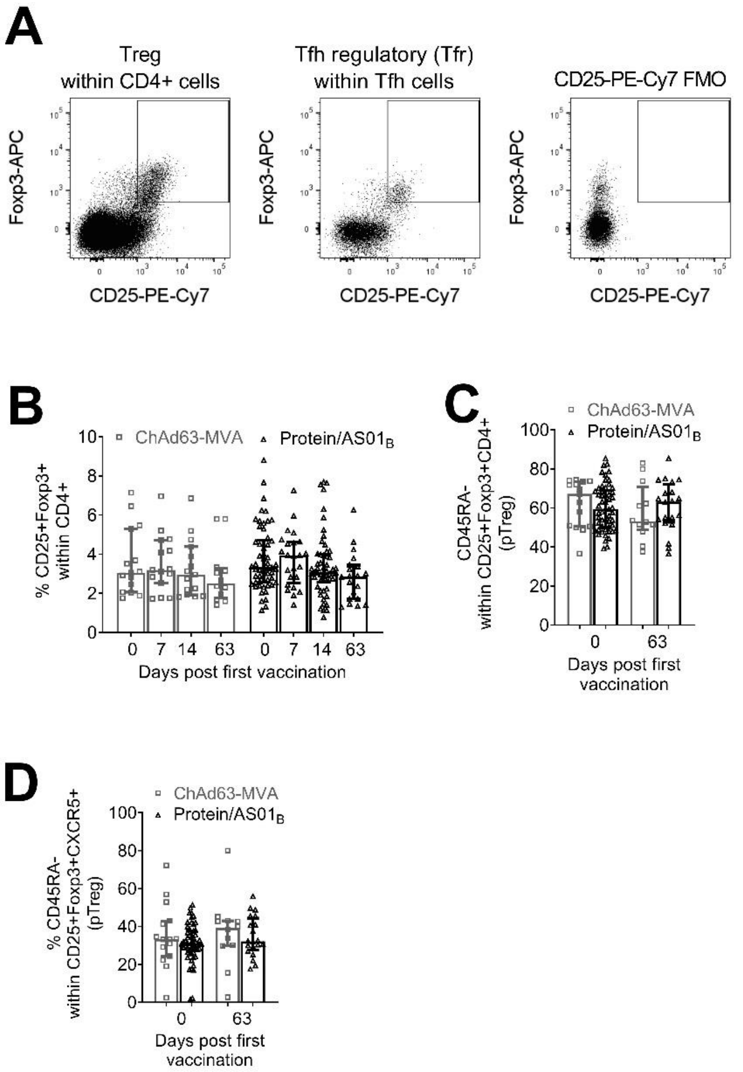

Supplemental Figure 3. Analysis of circulating CD4+ T regulatory (Treg) and T follicular regulatory (cTfr) phenotype cells in ChAd63-MVA and protein/AS01$_B$ vaccinees. Related to Figure 1. PBMC from Day 0, 7, 14, and 63 (one week following final vaccination) from vaccinees receiving PfRH5 delivered by ChAd63-MVA or protein/AS01$_B$ platforms were stained *ex vivo* for phenotypic markers and analyzed by flow cytometry. **(A)** Gating strategy for definition of CD25+Foxp3+ Treg cells within total CD4+ T cells and CD25+Foxp3+ cTfr cells within total cTfh cells. A leukapheresis cone sample from an unvaccinated donor stained with the full antibody panel apart from CD25-PE-Cy7 (fluorescence minus one [FMO]) is shown as a negative control. Total CD4+ T cell and CXCR5+ CD4+ cTfh cell gating strategies were as in Supplemental Figures 1 and 2, respectively, and CD45RA gating on Treg or cTfr cells was done as shown for total CD4+ T cells in Supplemental Figure 2. Frequencies of Treg cells within CD4+ T cells **(B)** and of CD45RA- peripherally-derived Treg (pTreg) cells within the Treg population **(C)** were compared within and between platforms. Similarly, the frequency of CD45RA- cells within the cTfr population **(D)** were compared within and between platforms. All available samples are plotted (ChAd63-MVA/ protein/AS01$_B$): Day 0, *n*=15/54; Day 7, n=15/24; Day 14, n=15/54; Day 63, *n*=12/20. For intra-trial comparisons **(B)** only vaccinees with all four time points were analyzed: ChAd63-MVA, *n*=12, protein/AS01$_B$, *n*=17. Comparisons were performed with either Mann-Whitney tests (between trials) or Friedman tests with Dunn's correction for multiple comparisons (within trials comparing Day 0 to post-vaccination time points). In all panels, each point represents a vaccinee. Bars and lines denote medians and interquartile ranges, respectively.

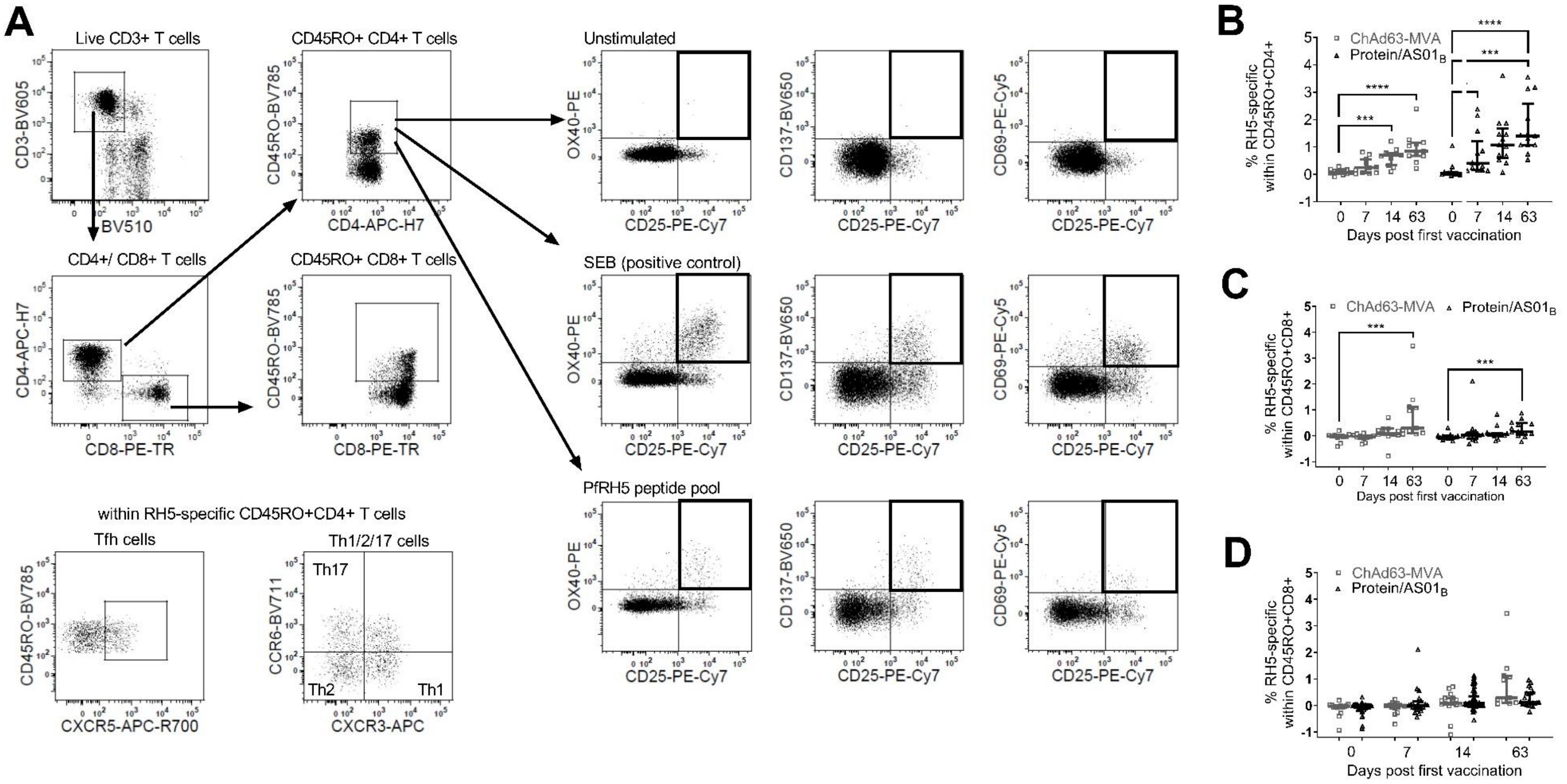

Supplemental Figure 4. Protein/AS01$_B$ platform induces a more robust PfRH5-specific CD4+ response than ChAd63-MVA vaccination. Related to Figures 2-3. PBMC from Day 0, 7, 14, and 63 (one week following final vaccination) from vaccinees receiving PfRH5 delivered by ChAd63-MVA or protein/AS01$_B$ were stimulated with medium alone, a PfRH5 peptide pool or SEB (positive control) for 24 hours, then stained and analyzed by flow cytometry. **(A)** Gating strategy for definition of: live CD3+ T cells (BV510 dump channel includes viability dye, anti-CD14 and anti-CD19) within single lymphocytes (as Figure 1), CD4+ and CD8+ T cells within total live T cells, CD45RO+ cells within total CD4+ or CD8+ T cell populations, and cells co-expressing CD25 with OX40, CD137 or CD69 following exposure to medium alone (unstimulated), SEB (positive control), or the PfRH5 peptide pool. Gating also shown on the third row for CXCR5+ Tfh cells and the frequencies of Th1 (CXCR3+CCR6-), Th2 (CXCR3-CCR6-) and Th17 (CXCR3-CCR6+) within the RH5-specific CD45RO+CD4+ T cells (total CD45RO+ CD4+ T cells responding to PfRH5 stimulation [CD25+OX40+ and/or CD25+CD69+ and/or CD25+CD137]). Frequencies of PfRH5-specific cells within CD45RO+CD4+ **(B)** or CD45RO+CD8+ **(C, D)** T cells were compared between Day 0 and each post-vaccination time point within trials **(B, C)** and at each time point between trials **(D)**. For intra-trial comparisons **(B, C)**, only vaccinees with all four time points available were analyzed: ChAd63, *n*=11, protein/AS01$_B$: *n*=13. For inter-trial analyses **(D)**, all available samples were analyzed (ChAd63-

MVA/ protein/$AS01_B$): Day 0, *n*=15/57; Day 7, *n*=15/20; Day 14, *n*=15/ 57; Day 63, *n*=11/22. Comparisons between trials were performed using Mann Whitney tests and comparisons within trials between Day 0 and each post-vaccination time point were performed with Friedman tests with Dunn's correction for multiple comparisons. *** $p<0.001$, **** $p<0.0001$. In all panels, each point represents a vaccinee. Lines denote medians and interquartile ranges.

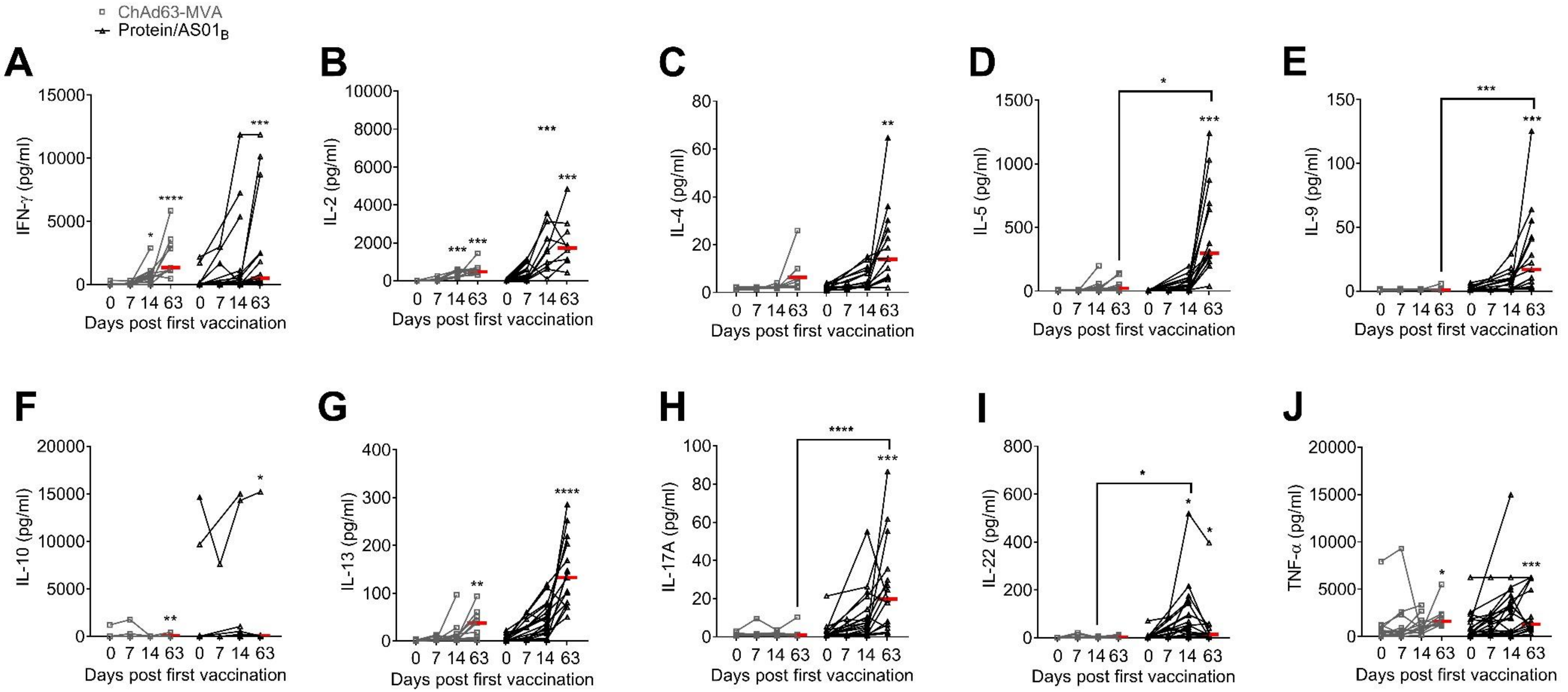

Supplemental Figure 5. Cytokine production following *in vitro* stimulation of PBMCs with PfRH5 peptides is skewed to a Th1 response in ChAd63-MVA vaccinees and towards Th2/Th17 in protein/AS01$_B$ vaccinees. Related to Figure 2. PBMC from Day 0, 7, 14, and 63 (one week following final vaccination) from vaccinees receiving PfRH5 delivered by ChAd63-MVA or protein/AS01$_B$ were stimulated with a PfRH5 peptide pool (2.5μg/ml for each peptide in the pool) for 24 hours. A multiplex bead-based assay was then used to measure supernatant concentrations of cytokines including **(A)** IFN-γ, **(B)** IL-2, **(C)** IL-4, **(D)** IL-5, **(E)** IL-9, **(F)** IL-10, **(G)** IL-13, **(H)** IL-17A, **(I)** IL-22, and **(J)** TNF-α. This assay was run on a subset of samples (ChAd63-MVA/ protein/AS01$_B$): Day 0, *n*=12/25; Day 7, n=12/12; Day 14, n=12/25; Day 63, *n*=9/15. Comparisons between trials were performed using Kruskal-Wallis tests and comparisons within trials between Day 0 and each post-vaccination time point were performed with paired Friedman tests, both with Dunn's correction for multiple comparisons. For intra-trial comparisons, only vaccinees with all four time points available were analyzed: ChAd63, *n*=9, protein/AS01$_B$: *n*=9. Statistics marked without brackets refer to intra-trial comparisons to Day 0. * p<0.05, ** *p*<0.01, *** *p*<0.001, **** *p*<0.0001. In all panels, each point represents a vaccinee. Bars and lines denote medians and interquartile ranges, respectively. Day 63 medians are emphasized with red lines.

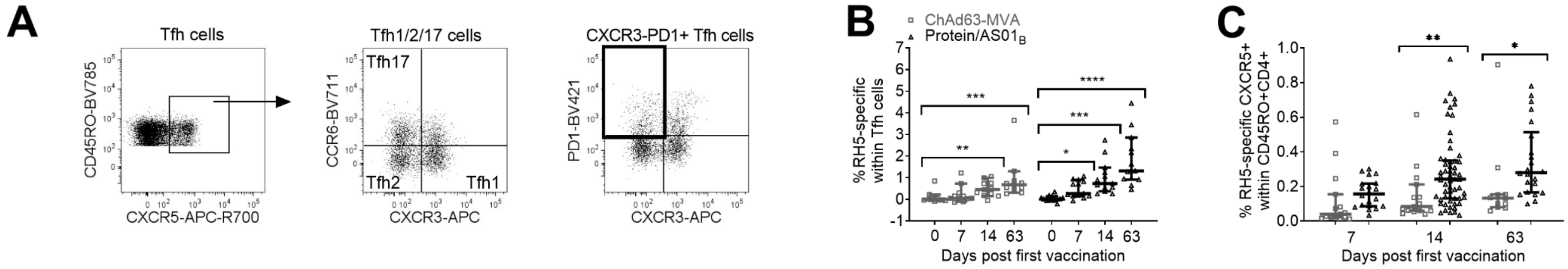

Supplemental Figure 6. Protein/AS01$_B$ platform induces a robust PfRH5-specific response within the cTfh cell population. Related to Figure 3. PBMC were stimulated as in Figure 4. **(A)** Gating strategy used to define cTfh cells as CXCR5+ cells within CD45RO+ CD4+ T cells, identify Tfh1/2/17 cells within the cTfh cell population based on CXCR3/CCR6 expression and delineate the CXCR3-PD1+ subset of CXCR5+CD45RO+CD4+ cTfh. Responding cells were defined based on co-expression of CD25+OX40+ and/or CD25+CD69+ and/or CD25+CD137+ (gated as shown in Supplemental Figure 4). **(B).** Frequencies of PfRH5 specific cells were compared within trials at each time point within cTfh cells. Only vaccinees with all four time points available were analyzed: ChAd63, *n*=11, protein/AS01$_B$: *n*=13. **(C)** Frequencies of PfRH5-specific Tfh cells within total CD45RO+CD4+ T cell populations were also compared between trials at post-vaccination time points. All available samples were analyzed (ChAd63-MVA/ protein/AS01$_B$): Day 7, *n*=15/20; Day 14, *n*=15/ 57; Day 63, *n*=11/22. Comparisons within trials were performed with Friedman tests with Dunn's correction for multiple comparisons **(B)**, and comparisons between trials were performed using Mann Whitney tests **(C)**. * $p< 0.05$, ** $p<0.01$, *** $p<0.001$, **** $p<0.0001$. Each point represents a vaccinee. Lines denote medians and interquartile ranges.

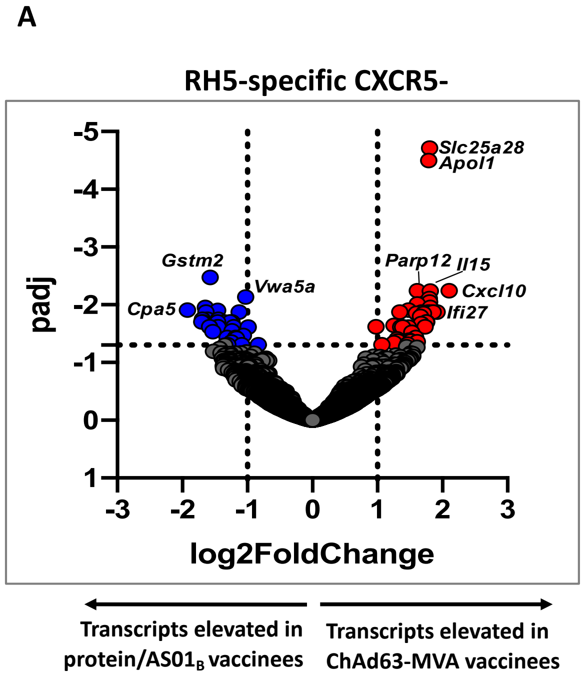

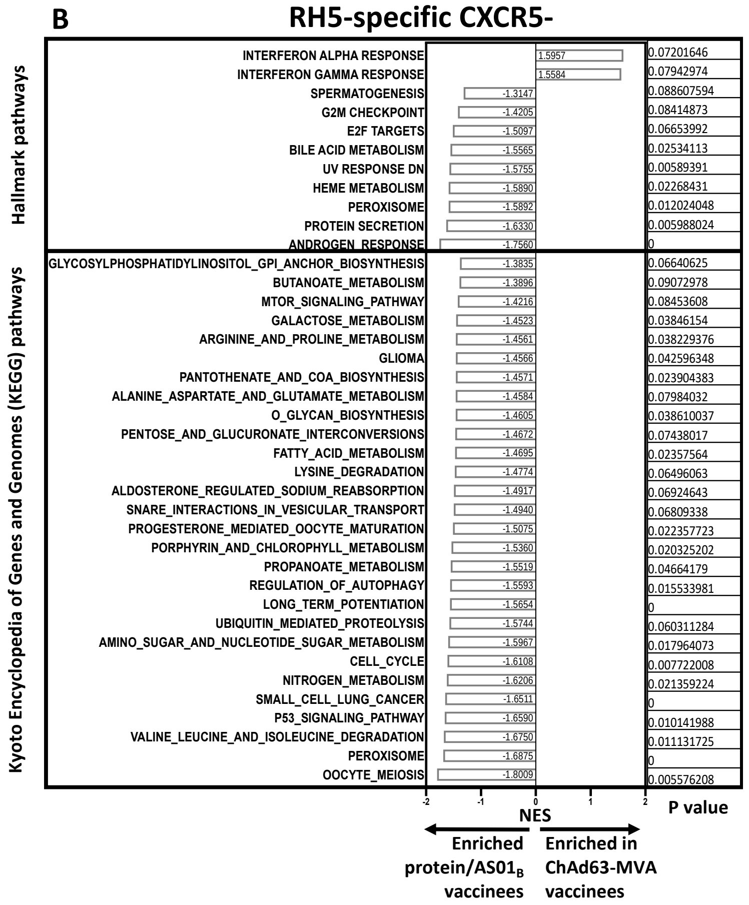

Supplemental Figure 7. Qualitative differences in the PfRH5-specific CXCR5- CD4+ T cells elicited by ChAd63-MVA versus protein/AS01$_B$ vaccines. Related to Figure 4. (A) Volcano plot illustrating genes that were differentially expressed in PfRH5-specific CXCR5- CD4+ T cells from ChAd63-MVA versus protein/AS01$_B$ vaccinees. FDR adjusted *p* values are shown on the y-axis and log(2) fold-change on the x-axis. Individual transcript names are shown for some points. **(B)** Bar chart showing results from gene ontology enrichment analysis using Hallmark (top) and KEGG (bottom) databases. The network enrichment score (NES) for each pathway enriched or trending to be enriched (p<0.1) in PfRH5-specific CXCR5+ cells from either ChAd63-MVA or protein/AS01$_B$ vaccinees is illustrated, together with the *p* value. RH5-Sp = RH5-specific.

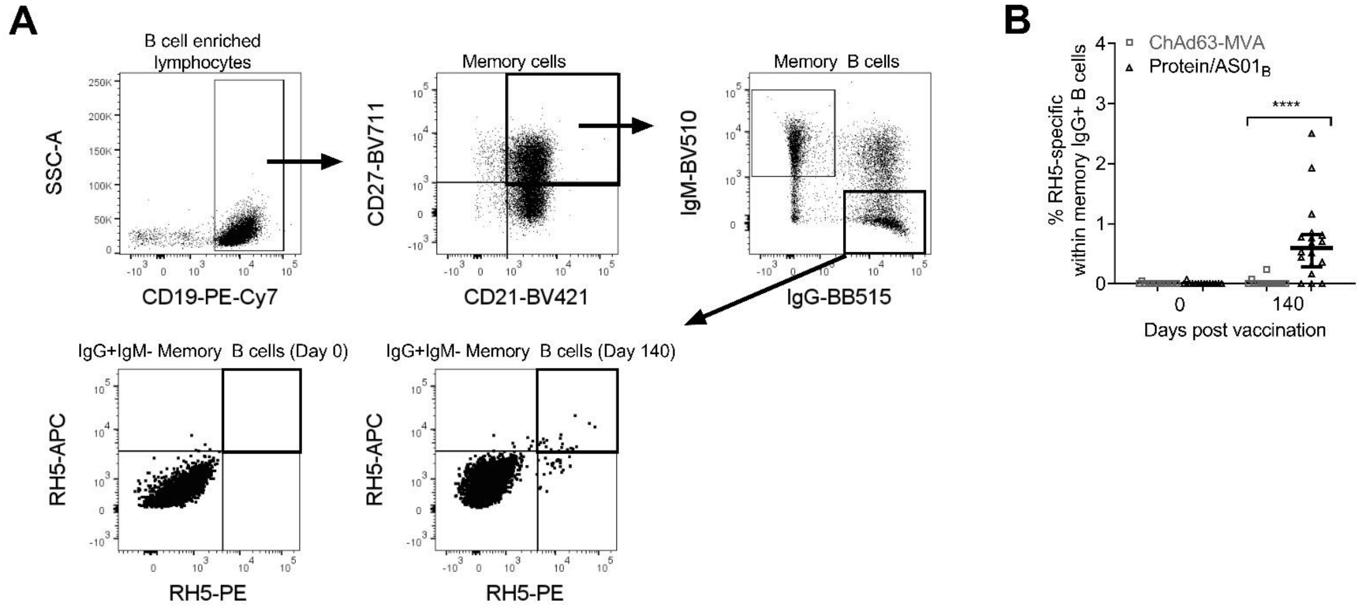

Supplemental Figure 8. Direct detection of PfRH5-specific memory B cells through co-staining with two PfRH5 probes. Related to Figure 5. PBMC from Day 0 and 140 (12 weeks after final vaccination) were enriched for B cells and then stained with phenotypic markers and analyzed by flow cytometry. **(A)** Gating strategy for definition of PfRH5-specific memory IgG+ B cells as CD19+CD21+CD27+IgG+IgM- cells within the live lymphocyte population that co-stained for monobiotinylated-PfRH5 conjugated to streptavidin-PE (PfRH5-PE) and monobiotyinlated-PfRH5 conjugated to streptavidin-APC (PfRH5-APC). Large dot settings have been used for clarity on the PfRH5 probe plots. PfRH5-specific memory B cells were compared between platforms at Day 0 and Day 140 using Mann Whitney tests **(B)**. **** $p<0.0001$. In panel B, each point represents a vaccinee. Lines denote medians and interquartile ranges.

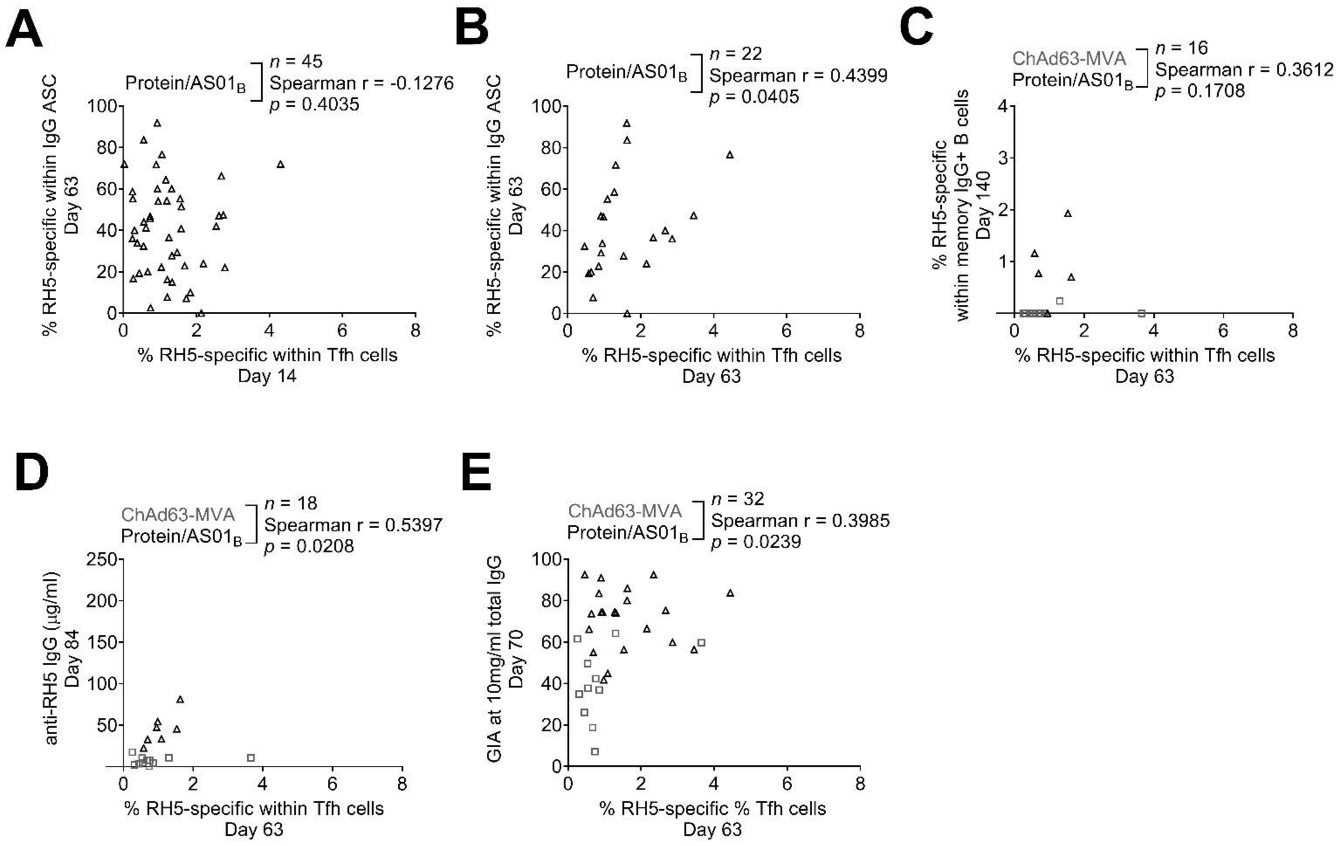

Supplemental Figure 9. cTfh cell responses and parameters of Th1:Th2 skew correlate with key markers of humoral immunogenicity. Related to Figure 5. Spearman correlation analyses were performed to interrogate the relationships between the frequency of PfRH5-specific cTfh cells at Day 14 **(A,)** or Day 63 **(B-E)** and the frequency of IgG antibody secreting cells (ASC) at Day 63 that were PfRH5-specific by IgG ELISPOT **(A, B)**, the frequency of IgG+ mBCs that were PfRH5-specific at Day 140 **(C)**, the serum anti-PfRH5 IgG concentration at Day 84 **(D)** and the purified IgG GIA at 10mg/ml at Day 70 **(E)**. Sample sizes, Spearman r, and *p* values annotated on the graphs refer analyses of pooled samples from both vaccine platforms (with the exception of **A/B** as IgG ASC ELISPOT data were only available for protein/$AS01_B$ vaccinees). In all panels, each point represents a vaccinee.

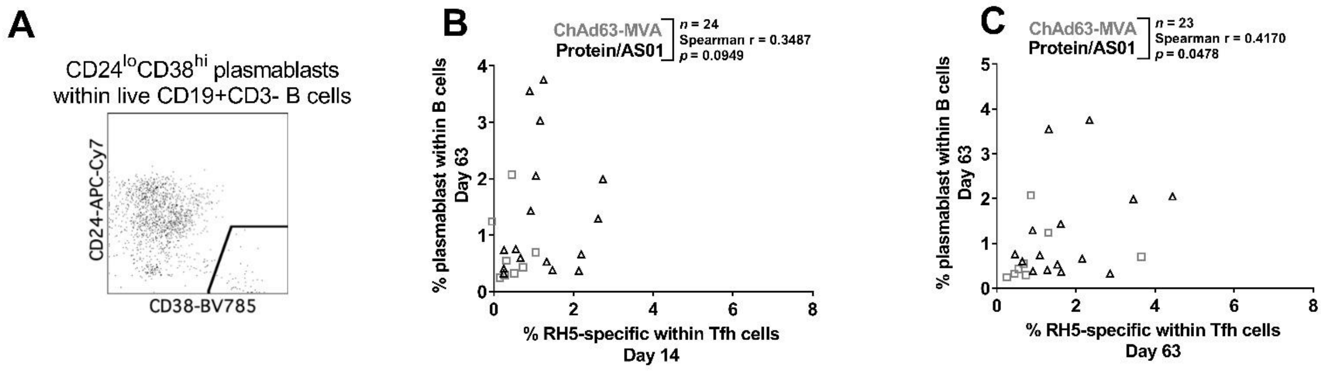

Supplemental Figure 10. Plasmablast frequency positively correlates with PfRH5-specific Tfh cells. Related to Figure 5. PBMC from Day 63 (one week following final vaccination) from vaccinees receiving PfRH5 delivered by ChAd63-MVA or protein/$AS01_B$ were stained *ex vivo* for B cell phenotypic and activation markers and analyzed by flow cytometry. **(A)** Representative flow cytometry plot from a vaccinee in the protein/$AS01_B$ trial showing plasmablast definition as CD24loCD38hi cells within live CD19+CD3- B cells. Spearman correlation analyses were performed to interrogate the relationship between the frequency of total plasmablasts at Day 63 and the frequency of PfRH5-specific cells within CD4+ Tfh cells (detected by co-expression of CD25 with OX40/ CD137/ CD69, Figure 4) at Day 14 **(B)** and Day 63 **(C).** Sample sizes, Spearman r, and *p* values annotated on the graphs refer to analyses of pooled samples from both vaccine platforms. In all panels, each point represents a vaccinee.

Supplemental Table 1. Genes exhibiting the significant differential expression in PfRH5-specific CXCR5- CD4+ T cells from ChAd63-MVA versus protein/$AS01_B$ vaccinees. Related to Table 2.

Genes significantly elevated in PfRH5-specific CXCR5- cells from ChAd63-MVA vs protein/$AS01_B$ vaccinees (padj< 0.05)			
**Gene Name**	**log2 FoldChange [a]**	**p value [b]**	**padj [c]**
SLC25A28	1.801339837	1.34E-09	1.94E-05
APOL1	1.787569019	4.39E-09	3.18E-05
CXCL10	2.103488763	1.92E-06	5.69E-03
PARP12	1.614413167	1.97E-06	5.69E-03
IL15	1.810696668	2.39E-06	5.76E-03
NFIX	1.797643815	4.34E-06	7.84E-03
LAMP3	1.801061212	5.59E-06	8.98E-03
HAPLN3	1.613517356	6.75E-06	9.76E-03
TBX21	1.81068457	9.31E-06	1.12E-02
TAP1	1.478880756	1.20E-05	1.24E-02
IFI27	1.915293007	1.51E-05	1.33E-02
LIF	1.858446264	1.67E-05	1.33E-02
ART3	1.677060328	1.76E-05	1.33E-02
ODF3B	1.344293158	1.91E-05	1.33E-02
CD33	1.81811709	2.04E-05	1.33E-02
B4GALT2	1.777312967	2.07E-05	1.33E-02
IRF1	1.597516912	2.34E-05	1.41E-02
JUND	1.708269826	2.46E-05	1.42E-02
ICAM1	1.661802109	2.86E-05	1.59E-02
OAS3	1.757923576	4.55E-05	1.99E-02
ZBP1	1.754828185	4.80E-05	1.99E-02
MCHR2	1.673927655	5.59E-05	2.13E-02
NAPA	1.254454753	6.15E-05	2.28E-02
CD274	1.386654414	6.85E-05	2.42E-02
IRG1	1.733076316	7.17E-05	2.42E-02
GCH1	0.978495033	7.51E-05	2.42E-02
IFI35	1.479185105	7.99E-05	2.44E-02
GBP4	1.358810904	8.25E-05	2.44E-02
RBCK1	1.387930926	8.46E-05	2.45E-02
ETV7	1.527106033	1.10E-04	2.99E-02
TYMP	1.586185479	1.45E-04	3.69E-02
APOL2	1.303897925	1.59E-04	3.89E-02
LGALS9	1.485169083	1.71E-04	4.11E-02
VWA7	1.512502476	1.79E-04	4.25E-02
FBXO6	1.311412591	1.82E-04	4.25E-02
PTGDR2	1.61460337	1.91E-04	4.39E-02
LINC00174	1.250029755	2.04E-04	4.55E-02
EPSTI1	1.461310157	2.20E-04	4.81E-02
FOXK2	1.065546525	2.40E-04	4.95E-02

Genes significantly reduced in pfRH5 specific CXCR5- cells from ChAd63-MVA vs protein/$AS01_B$ vaccinees (padj< 0.05)			
**Gene Name**	**log2 FoldChange [d]**	**p value [b]**	**padj [c]**
GSTM2	-1.574644968	7.01E-07	3.38E-03
VWA5A	-1.027818154	3.55E-06	7.34E-03
ABCD2	-1.645183878	9.00E-06	1.12E-02
CPA5	-1.921288731	1.19E-05	1.24E-02
LYPD3	-1.455678814	1.30E-05	1.25E-02
TEPP	-1.638467221	2.05E-05	1.33E-02
TC2N	-1.127301624	2.12E-05	1.33E-02
TMIGD2	-1.445500444	3.34E-05	1.78E-02
FBLN5	-1.69183961	3.51E-05	1.78E-02
DEPDC7	-1.614112062	3.57E-05	1.78E-02
TTC3	-1.269272703	4.54E-05	1.99E-02
STMN1	-1.359827123	4.75E-05	1.99E-02
TXK	-1.455403726	4.78E-05	1.99E-02
PRKACB	-1.237697265	4.93E-05	1.99E-02
C2orf40	-1.701577155	5.04E-05	1.99E-02
PGLYRP2	-1.683339766	5.10E-05	1.99E-02
HERC2P9	-1.211339677	6.84E-05	2.42E-02
NUCB2	-1.456536663	7.44E-05	2.42E-02
CENPK	-1.584143966	7.70E-05	2.42E-02
SPICE1	-1.453705336	7.70E-05	2.42E-02
ADD3	-0.985979725	8.17E-05	2.44E-02
SIRPG	-1.239087746	1.00E-04	2.84E-02
CEP152	-1.532159776	1.06E-04	2.94E-02
NDRG2	-1.06374275	1.28E-04	3.42E-02
CAMK2D	-1.183049451	1.43E-04	3.69E-02
PAIP2B	-1.310415505	1.46E-04	3.69E-02
ZFP14	-1.165064786	1.53E-04	3.82E-02
TMEM2	-1.249189039	1.97E-04	4.46E-02
FZD3	-0.833137043	2.32E-04	4.95E-02
ZNF404	-1.089966388	2.35E-04	4.95E-02
HLTF	-1.303895632	2.39E-04	4.95E-02

a: Elevation in ChAd63-MVA versus protein/$AS01_B$ vaccinees

b: Difference between groups, unadjusted *p* value

c: False discovery rate (FDR) adjusted *p* value. Only genes for which padj<0.05 are listed.

d: Reduction in ChAd63-MVA versus protein/AS01 vaccinees

Supplemental Table 2. Summary of Tfh2/Th1/Th2 parameter correlations with humoral immunogenicity read-outs. Related to Figure 5.

**Read-out**	**ASC (% RH5-specific within IgG ASC, Day 63)**	**IgG (Day 84 anti-RH5 IgG μg/mL)**	**Memory B cell (% RH5-specific within memory IgG+ B cells, Day 140)**	**GIA (Day 70 GIA at 10mg/mL IgG)**
**Th1 (% Th1 within RH5-specific CD4+ T cells, Day 14)**	*n* = 44<br>Spearman r = 0.1964<br>*p* = 0.2014	***n* = 44**<br>**Spearman r = -0.3710**<br>***p* = 0.0132**	***n* = 29**<br>**Spearman r = -0.4084**<br>***p* = 0.0278**	*n* = 57<br>Spearman r = -0.06332<br>*p* = 0.6398
**Th2 (% Th2 within RH5-specific CD4+ T cells, Day 14)**	*n* = 44<br>Spearman r = -0.02841<br>*p* = 0.8548	***n* = 44**<br>**Spearman r = 0.3458**<br>***p* = 0.0215**	*n* = 29<br>Spearman r = 0.3362<br>*p* = 0.0746	***n* = 57**<br>**Spearman r = 0.4215**<br>***p* = 0.0011**
**Tfh2 (% Th2 within RH5-specific Tfh cells, Day 14)**	*n* = 28<br>Spearman r = 0.1089<br>*p* = 0.5811	*n* = 24<br>Spearman r = 0.1261<br>*p* = 0.5572	***n* = 16**<br>**Spearman r = 0.5194**<br>***p* = 0.0415**	*n* = 33<br>Spearman r = 0.2263<br>*p* = 0.2054

ASC = antibody secreting cell. PfRH5-specific defined by co-staining with PfRH5-PE and PfRH5-APC probes (see Supplemental Figure 8). GIA = growth inhibitory activity. Th1, Th2, and Tfh2 read-outs are from flow cytometry-based analyses of the AIM assay, while cytokine ratios are calculated from Th cytokine concentration measurements from supernatants from the same assay. Purple cells highlight negative correlations and green cells highlight positive correlations. Bold text indicates statistically significant correlations at $\alpha = 0.05$ level.

Supplemental Table 3. Antibodies used in *ex vivo* and *in vitro* panels. Related to STAR Methods.

Panel and Relevant Figures	Anti-human antibody	Clone	Cat #	Supplier
Ex vivo T cells<br><br>**Figure 1**<br>**Supplemental Figures 1-3**	CXCR5-BB515	RF8B2	564624	BD
	CXCR3-PE-Cy5	1C6/CXCR3	551128	BD
	CD25-PE-Cy7	2A3	335824	BD
	CD3-PE-TR	7D6	MHCD0317	Life Tech.
	CCR6-PE	11A9	551773	BD
	CD4-APC-H7	SK3	641398	BD
	CD8a-AF700	RPA-T8	301028	Biolegend
	CD38-BV785	HIT2	303530	Biolegend
	CD45RA-BV711	HI100	304138	Biolegend
	CCR7-BV650	G043H7	353234	Biolegend
	ICOS-Biotin	ISA-3	13-9948-82	Invitrogen
	PD1-BV421	EH12.2H7	329920	Biolegend
	Live/Dead Aqua*	n/a	L34966	Invitrogen
	Streptavidin-BV605*	n/a	563260	BD
	Ki67-PerCP-ef710	20Raj1	46-5699-42	Invitrogen
	Foxp3-APC	PCH101	17-4776-42	eBioscience
In vitro T cells<br><br>**Figures 2-5**<br>**Supplemental Figures 4, 6-7, 9**	CD183-APC	1C6/CXCR3	550967	BD
	CXCR5 APC-R700	RF8B2	565191	BD
	ICOS-Biotin	ISA-3	13-9948-82	Invitrogen
	CCR6-BV711	G034E3	353436	Biolegend
	PD1-BV421	EH12.2H7	329920	Biolegend
	Live/Dead Aqua*	n/a	L34966	Invitrogen
	CD14- BV510	M5E2	301842	Biolegend
	CD19-BV510	SJ25C1	562947	BD
	CD137-BV650	4B4-1	309828	Biolegend
	CD45RO-BV785	UCHL1	304234	Biolegend
	CD4-APC-H7	SK3	641398	BD
	CD39-PerCP-ef710	eBioA1	46-0399-42	eBioscience
	Streptavidin-BB515*	n/a	564453	BD
	OX40-PE	L106	340420	BD

	CD69-PE-Cy5	FN50	555532	Biolegend
	CD25-PE-Cy7	2A3	335824	BD
	CD8-PE-TR	3B5	MHCD 0817	Invitrogen
	CD3-BV605	UCHT1	300460	Biolegend
***Ex vivo* B cells**<br><br>**Figure 5**<br>**Supplemental Figures 8-9**	CD19-PE-Cy7	SJ25C1	557835	BD
	IgG-BB515	G18-145	564581	BD
	CD27-BV711	M-T271	564893	BD
	CD21-BV421	B-ly4	562966	BD
	IgM-BV510	G20-127	563113	BD
	Fixable viability stain 780*	n/a	565388	BD
	Monobiotinylated RH5*	n/a, produced in-house		
	Streptavidin-PE*	n/a	S866	Invitrogen
	Streptavidin-APC*	n/a	405207	eBioscience
***Ex vivo* B cells**<br><br>**Supplemental Figure 10**	CD21-FITC	Bu32	354910	Biolegend
	IgD-PE-Cy7	IA6-2	348210	Biolegend
	CD3-PE-TR	7D6	MHCD0317	Life Tech.
	LAP-PE	27232	FAB2463P	R&D Systems
	CD24-APC-H7	ML5	311132	Biolegend
	CD40-AF700	5C3	334328	Biolegend
	CD38-BV785	HIT2	303530	Biolegend
	CD27-BV711	O323	302834	Biolegend
	CD19-BV650	HIB19	302238	Biolegend
	CD43-BV421	1G10	562916	BD
	Live/Dead-Aqua*	n/a	L34966	Invitrogen
	PDL1-PerCP-ef710	MIH1	46-5983-42	Ebioscience
	PDL2-PerCP-ef710	MIH18	46-5888-42	Invitrogen
	IgM-AF647	MHM-88	314535	Biolegend

Cells shaded in grey indicate those antibodies which were not used in the analyses presented in this report.
n/a = not applicable
* Not anti-human antibodies
